# Chitosan-titanium oxide fibers supported zero-valent nanoparticles: Highly efficient and easily retrievable catalyst for the removal of organic pollutants

**DOI:** 10.1038/s41598-018-24311-4

**Published:** 2018-04-19

**Authors:** Fayaz Ali, Sher Bahadar Khan, Tahseen Kamal, Khalid A. Alamry, Abdullah M. Asiri

**Affiliations:** 1 Center of Excellence for Advanced Materials Research (CEAMR), King Abdulaziz University, P.O. Box 80203, Jeddah, 21589 Saudi Arabia; 2 Department of Chemistry, King Abdulaziz University, P.O. Box 80203, Jeddah, 21589 Saudi Arabia

## Abstract

Different chitosan-titanium oxide (CS-TiO_2_-x, with x = TiO_2_ loadings of 1, 5, 10,15 and 20 wt%) nanocomposite fibers were prepared and kept separately in each salt solution of CuSO_4_, CoNO_3_, AgNO_3_ and NiSO_4_ to adsorb Cu^2+^, Co^2+^, Ag^+^, and Ni^+^ ions, respectively. The metal ions loaded onto CS-TiO_2_ fibers were reduced to their respective zero-valent metal nanoparticles (ZV-MNPs) like Cu^0^, Co^0^, Ag^0^ and Ni^0^ by treating with NaBH_4_. The CS-TiO_2_ fibers templated with various ZV-MNPs were characterized and investigated for their catalytic efficiency. Among all prepared ZV-MNPs, Cu^0^ nanoparticles templated on CS-TiO_2_-15 fibers exhibited high catalytic efficiency for the reduction of dyes (methyl orange (MO), congo red (CR), methylene blue (MB) and acridine orange (AO)) and nitrophenols (4-nitrohphenol (4-NP), 2-nitrophenol (2-NP), 3-nitrophenol (3-NP) and 2,6-dinitrophenol (2,6-DNP)). Besides the good catalytic activities of Cu/CS-TiO_2_-15 fibers, it could be easily recovered by simply pulling the fiber from the reaction medium.

## Introduction

Metals nanoparticles (MNPs) are receiving more attention due to their specificity of interaction and relatively higher chemical activity as compared to their bulk states. Efforts in research have been increased to prepare the nano-scale material for the detection and detoxification of the polluted water from different types of water bodies^1–3^. The key for selective and enhance activity is to control the size, shape and dispersity of the nanoparticles. The other importance for rapid developing in the field of nanoparticles preparation is the remarkable differing physico-chemical properties of MNPs as compared to their bulk equivalents. MNPs have large surface to volume ratio which make them attractive for many applications^4^. Zero-valent MNPs are considered as strong reducing agent due to their electron-donating tendency and have been widely used for removal of different kind of contaminants including the anions reductions^5,6^. These MNPs also catalyze the degradation of chemical pollutants such as nitrobenzene, nitrophenols, chlorinated phenols, chlorohydrocarbon, polychlorinated biphenyls from waste water, drinking water and ground water due to its great surface reactivity, large surface area and relatively low price^7–10^. In addition to these extraordinary properties, nanoparticles characteristically provide highly active centers but are immense small and are not stable thermodynamically. At this size regime, structures are highly unstable (agglomerates or oxidize in air) due to their extra-large surfaces and high surface energy^11^. This can lead to the decline in reactive site as well as adsorption sites of nanoparticles, resulting in the decrease in contact chances between MNPs and target pollutants^12^. For stable particle production, the control of particle growth reaction is necessary. A number of methods have been applied to achieve this goal, for example addition of other metal salts or organic ligand, or by inorganic capping materials, or by colloids and soluble polymer as well as creating core shell type particle morphology^13,14^. However, problem arises with respect to reuse and efficient activity of these catalysts, as the nanoparticles possibly will experience aggregation and suffer from harming under the reaction circumstances, resulting in loss of catalytic activity and deactivation. Large number of research articles have been published with the stated aim of producing highly active nanoparticles and other nanomaterials with consistent size, dispersity and inhibiting aggregation for the decontamination of water^15,16^. In this regard, metallic nanoparticles are supported within the matrices of a variety of solid supports^2,4,17–27^. The fusion of nanoparticle technology and porous materials is potentially one of the most stimulating and fruitful areas of research. Over the past decade, such supported MNPs materials have attracted much attention due to the potential of the size/shape selective catalysis and the potential for enhanced selectivity, control of the size, shape and activity of the nanoparticle, as well as the advantage of inhibition of the nanoparticle aggregation provided by separation/immobilization on the heterogenous support^3^.

Normally, a porous material is a solid composed of unified network of pores (voids). A variety of porous materials have been exploited for the controlled synthesis of metallic nanoparticles. Each support has its own advantages. Some of the most commonly applied supports for the deposition of MNPs are polymers, carbonaceous materials and metal oxides^3^. Recently the reported supports for zero valent MNPS are SiO_2_^5^, boron nitride nanoplates^28^, zeolites^29,30^, graphene^12^, biochar^31^, carbon nanotube^32^, chitosan^18,20,33–37^, chelating resin^6^. Polymer, chitosan is mostly selected as a support for MNPs because of its wide availability, low cost, nontoxicity, biodegradability and unique structural possessions. The presence of reactive amino group on the backbone enable chitosan for the addiction of several metal ions. The metal sorbs on chitosan through several mechanisms including electrostatic interaction (such as the formation of ion pair or ion-exchange) and chemical interaction with hydroxyl and amine group of chitosan. However, using only chitosan as a support is not able to sorb that much of metal ions because of its weak mechanical strength and weak chemical properties (dissolve in acid solution). Therefore, to improve the performance of chitosan as a support for MNPs, the modification in chemical and physical properties is necessary. Different methods were applied for its modification, where chitosan coating on common substances is a new method. The use or mixing of proper and commercially available materials with chitosan will improve its mechanical and chemical properties. Recently, different kind of materials such as clay mineral montmorillonite^38–40^, cellulose filter paper^18,41^, cellulose microfibers^4,17^, carbon nanotube^42^, or inorganic nanoparticles^2,43–45^, among others, have been used to improve the properties of chitosan for various applications. Among the various kinds of inorganic nanoparticles, TiO_2_ nanoparticles are of intense interest due to their ability to advances mechanical properties of chitosan as well as to improve its antibacterial activity, good chemical stability, biocompatibility, photocatalytic activity, UV shielding ability, nontoxicity and low price^46,47^. In literature, several reported studies elaborate the formation of chitosan/TiO_2_ nano-biocomposites and their characterization in terms of mechanical, optical, thermal and antibacterial properties^44,48–51^. It is reported that composite material containing TiO_2_ with chitosan showed high mechanical reinforcement, *i.e* addition of 30% TiO_2_ (by weight relative to chitosan) resulted an increase of 11.8 fold in Young’s modulus, a 6 fold increase in toughness and a 6 fold increase in tensile strength^48^. Similarly, in other study it is reported that the thermal stability was improved with the addition of TiO_2_ to chitosan matrix^49^. It has been reported that CS-TiO_2_ nanocomposite lacks the health risks such as transport to the body and biodegradability and specific antibacterial property toward bone-infecting microorganisms^50^. One of the studies reported that fabrication of TiO_2_ with chitosan exhibited good photocatalytic activity for dyes degradation^51^. However, to our knowledge the fabrication of TiO_2_ with chitosan as a support of MNPs used for the remediation of organic pollutants present in wastewater has not been reported yet.

Presence of organic pollutants such as nitrophenols and organic dyes in water are considered as toxic and pose a risk to the human health, exposure to these contaminates only for a short time causing eye and skin irritation, confusion, unconsciousness, convulsions, cough, dizziness, headache, nausea, sore throat, abdominal pain, vomiting, etc^52^. Dye and nitrophenols degradation or removal from wastewater has been widely investigated to reduce their influence on the environment. Among various treatment methods, catalytic reduction of azo dyes and nitrophenols in the presence of sodium borohydride (NaBH_4_) has been widely investigated^53–60^. The catalytic conversion of nitrophenol has become one of the model reactions because of its complete conversion without production of by-products and easy measurement for both nitrophenol (reactant) and aminophenol (product) by using UV-vis spectroscopy^61^.

Keeping in mind marvelous properties of nano zero valent metal particles and TiO_2_ embedded with chitosan, we prepared supported MNPs on the surface of CS-TiO_2_ composite fibers for removal of organic pollutants present in wastewater.

## Experimental

### Chemicals and reagents

Chitosan having degree of deacetylation >75% and with high molecular weight (800–2000 cP), was purchased from Sigma Aldrich, Ireland. Sodium hydroxide, sodium borohydride and salts of copper sulphate, nickel sulphate, cobalt nitrate and silver nitrate were also bought from Sigma-Aldrich. Acetic acid was obtained from NTN Ltd, U.K. The required reagents such as nitrophenols (3-nitrophenol (3-NP), 2-nitrophenol (2-NP), 4-nitrophenol (4-NP) and 2,6-dinitrophenol (2,6-DNP)) and methylene blue (MB), acridine orange (AO), methyl orange (MO) and congo red (CR) were bought from BDH chemicals, England and other dyes were obtained from the chemical store in King Abdulaziz University. The chemical formulae of these pollutants are nitrophenols (C_6_H_5_NO_3_), 2,6-DNP (C_6_H_4_N_2_O_5_), MO (C_14_H_14_N_3_NaO_3_S), MB (C_16_H_18_ClN_3_S), AO (C_17_H_19_N_3_), CR (C_32_H_22_N_6_Na_2_O_6_S_2_). Titanium dioxide (TiO_2_) powder in anatase form was purchased from Sigma Aldrich. The particles size was <25 nm. The deionized water used for preparation of samples was obtained from departmental Millipore-Q water purification system having resistivity of 18.2 MΩ.

### *In-situ* preparation of supported metal nanoparticles (SMNPs)

#### Synthesis of titanium dioxide-chitosan (CS-TiO_2_) nanocomposite as a support

Chitosan powder was completely dissolved in 20% v/v aqueous acetic acid solution by overnight stirring. The 2 wt% of chitosan solution was used for the synthesis of chitosan-titanium dioxide nanocomposite (CS-TiO_2_). Different weight percentage (1 wt%, 5 wt%, 10 wt%, 15 wt% and 20 wt%) of TiO_2_ nanomaterials was taken, added and dispersed in chitosan solution through constant stirring. The composite fibers were prepared from that solution with the help of a clean disposable syringe in concentrated NaOH aqueous solution, in order to evaluate its performance for different applications. Then these composite fibers were removed from the NaOH solution, washed several times with water and finally dried at room temperature. The CS-TiO_2_ nanocomposite fibers containing 1, 5, 10, 15 and 20 wt% of TiO_2_ were represented as CS-TiO_2_-1, CS-TiO_2_-5, CS-TiO_2_-10, CS-TiO_2_-15, CS-TiO_2_-20, repectively.

#### Synthesis of metal nanoparticles on CS-TiO_2_ nanocomposite

**Figure 1:**
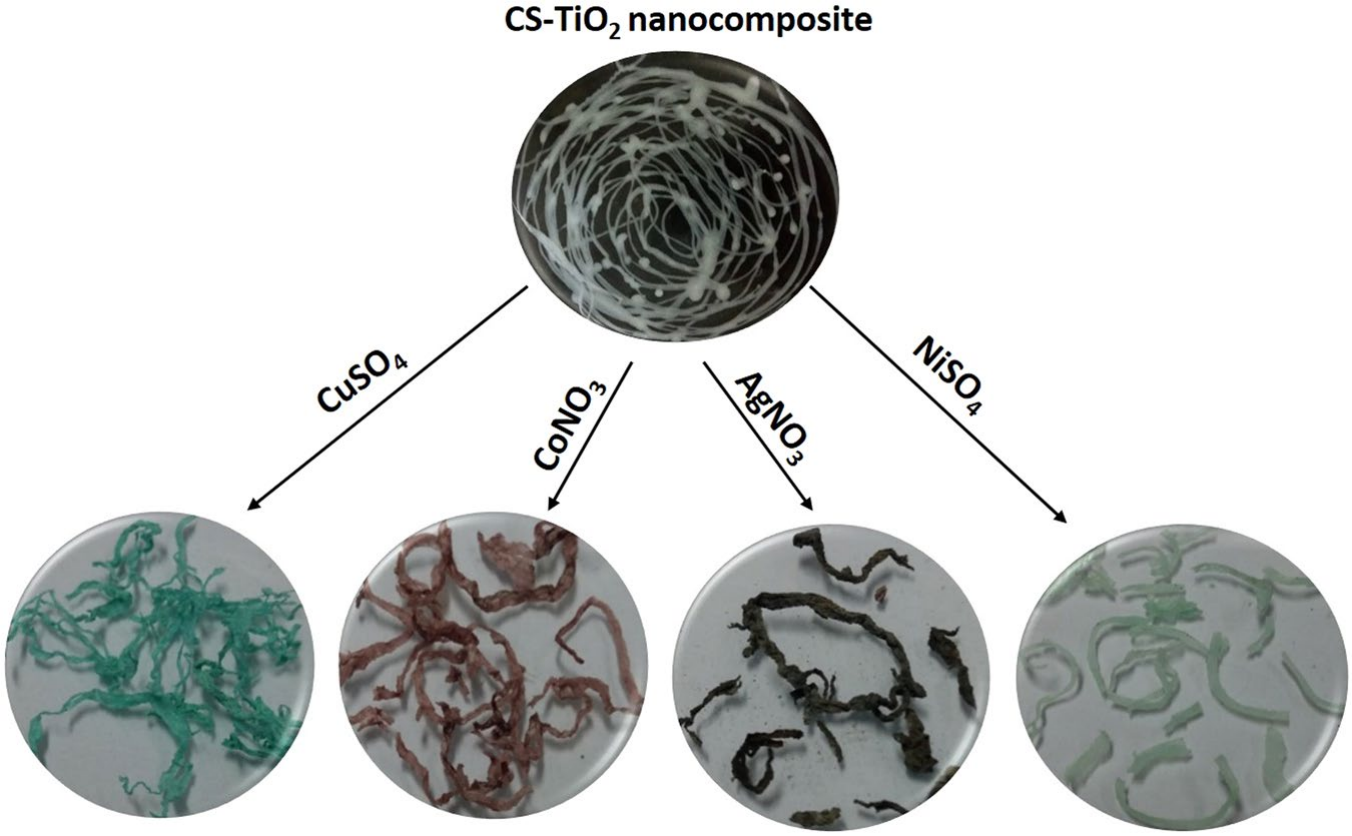
Pictures of pure CS-TiO_2_ and treated with respective metal salt solutions.

The different wt% of CS-TiO_2_ composite fibers were used as a support for the synthesis of MNPs. The following steps were followed for the synthesis of supported MNPs which are presented in Fig. 1. The dried CS-TiO_2_ (different wt% of TiO_2_) fibers were put in metal salt solutions for three hours, in order to uptake metals ions (step 1). In total, four metal ions containing solutions were prepared, in which, 0.1 M concentration of CuSO_4_, NiSO_4_, CoNO_3_ and AgNO_3_ salts were prepared in deionized water. The color change was observed by putting the CS-TiO_2_ (different wt%) fibers in these metal ions containing solutions. The color of the fibers become bluish, pinkish, brownish and greenish due to Cu^2+^, Co^2+^, Ag^+^ and Ni^+^ ions adsorption, respectively (Fig. 1). The treated CS-TiO_2_ (different wt% of TiO_2_) fibers with metal ions solutions were collected, dried and employed for the synthesis of nanoparticles by putting in freshly prepared 0.1 M NaBH_4_ aqueous solution (step 2). The respective color of all the metal ions loaded fibers become black as we dipped in the NaBH_4_ aqueous solution, which suggest that metal ions were converted to zero-valent MNPs. These fibers were kept in NaBH_4_ aqueous solution for 30 min, in order for complete reduction of metal ions into zero-valent nanoparticles as shown by the given equations

1 $${{\rm{M}}}^{2+}+4{{{\rm{BH}}}^{-}}_{4}+12{{\rm{H}}}_{2}{\rm{O}}\to 2{{\rm{M}}}^{0}+14{{\rm{H}}}_{2}+4{\rm{B}}{({\rm{OH}})}_{3}$$

2 $${{\rm{M}}}^{+}+2{{{\rm{BH}}}^{-}}_{4}+6{{\rm{H}}}_{2}{\rm{O}}\to {{\rm{M}}}^{0}+7{{\rm{H}}}_{2}+2{\rm{B}}{({\rm{OH}})}_{3}$$

Thus zero-valent MNPs (M^0^) templated on CS-TiO_2_ composite fibers were resulted from this treatment with NaBH_4_, where the respective metal ions of corresponding metal clusters were reduced to zero-valent metal nanoparticles (M^0^).

### Characterization of SMNPs

The morphology of the nanocomposite and SMNPs was investigated with the help of field emission scanning electron microscope (FE-SEM), of JEOL, JSM-7600F, Japan. All the samples were first coated with a thin layer of platinum in a vacuum chamber for 60 seconds before imaging. For SEM observation, the samples were stuck with the help of carbon tape onto a stub of 1 cm diameter. The SEM image was taken in secondary electron mode with a beam of energy 5.0 KV. Histograms of the nanostructure dimensions were obtained by further processing the SEM images using ImageJ software package. The oxford Energy dispersive spectrometer (oxford-EDS), equipped with SEM was used for the elemental analysis of the SMNPs.

Crystalline structure was analyzed by using x-ray diffraction (XRD) on PANalytical diffractometer with a Cu kα radiation source of wavelength, λ = 0.154 nm. The instrument was run at 40 kV and 40 mA and the data was obtained in 2θ range of 10° to 80° at a scan rate of 1° min^−1^ at room temperature.

The crystallite size was calculated from XRD pattern with the help of the following Scherrer equation,

3 $$\tau =\frac{k\lambda }{\beta cos\theta }$$

where τ represent crystallite size, λ is the wavelength of X-rays (1.548 A°), β exhibit the full width of the peak at half maximum (FWHM) and θ is the scattering angle of the peak. Fityk software was used for the calculation of FWHM from XRD pattern.

PerkinElmer ATR-FTIR spectrometer was used to record the spectra of pure and loaded nanocomposite fibers in the range of 400-4000 cm^−1^. The residual concentration of nitrophenols and dyes in catalytic reduction studies were monitored and measured with the help of Thermo Scientific Evolution 300 UV-visible spectrophotometer.

### Evolution of catalytic activity

The catalytic activity of the CS-TiO_2_ nanocomposite fibers with different wt% of TiO_2_ loaded with different MNPs were investigated for different chemical reactions. All these experiments were performed in dark conditions using UV-cuvette as reaction vessel which was placed in the dark compartment of the spectrophotometer. Among these catalytic reactions involved nitrophenols (3-NP, 2-NP, 4*-*NP and 2,6-DNP) conversion to aminophenols, and reduction of MB, AO, MO and CR. Also, we investigated the catalytic reduction of the solutions containing more than one dye or nitrophenol mix with dye. As an example, here we present the procedure for the conversion of 4-NP to 4-AP in the presence of NaBH_4_, the given representative procedure was executed for all the catalytic reduction reaction using the prepared SMNPs as a catalyst.

Deionized water was used to prepare the required solutions of NaBH_4_, nitrophenols and azo dyes having respective concentration 0.1 M, 0.1 mM and 0.05 mM. From the prepared solution of 4-NP (0.1 mM), 2.5 mL was taken in the cuvette, adding 0.5 mL of freshly prepared NaBH_4_ solution, in the meantime of addition of NaBH_4_, the color change was observed from bright yellow to dark yellow. The cuvette was put in UV-vis spectrophotometer to observe the absorbance spectra of 4-NP before and after the addition of NaBH_4_. Besides, the CS-TiO_2_ composite fibers with different wt% of TiO_2_ templated with different MNPs was added abruptly after the addition of NaBH_4_ to the cuvette containg 4-NP solution. In this way, the role of added TiO_2_ with different wt% and templated with different MNPs was investigated on the conversion of 4*-*NP to 4-AP. The regular decrease in the maximum absorbance (λ_max_) at 400 nm was observed for 4-NP and recorded after every 60 seconds by using UV-Vis spectrophotometer. The catalytic performance of the composite fibers loaded with different MNPs was determined with the following formula

4 $${\rm{Percent}}\,{\rm{conversion}}/{\rm{reduction}}=\frac{{{\rm{A}}}_{{\rm{o}}}-{{\rm{A}}}_{{\rm{t}}}}{{{\rm{A}}}_{{\rm{o}}}}\times 100$$

Where A_0_ is the initial absorbance of 4-NP at λ_max_, while A_t_ is the absorbance at different intervals of time (t).

Same procedure was used for the catalytic conversion of 2-NP, 3-NP and 2,6-DNP as well as for catalytic reduction of azo dyes (MB, AO, MO and CR). Besides, the same procedure was also used to investigate the catalytic reduction of solutions containing mixed dyes and nitrophenol with dye. Newly templated MNPs composite fibers were used as a catalyst in all the reaction except in the recyclability test of the composite catalyst where the same fiber was reused.

## Results and Discussion

### Schematic representation of SMNPs preparation

Figure 1 represents the step taken in the synthesis of SMNPs on composite fiber of CS-TiO_2_ with different wt% of TiO_2_. Recently, the metal ions uptake was investigated on nonoporous TiO_2_, and found good activity for the metal ions adsorption from aqueous system due to narrow and strong pore size distribution and high specific surface area^62^. It is also reported in another study that the use of TiO_2_ as nanofiller modify the molecular network of polymer and enhance the surface area for metal adsorption^63^. The nanocomposite of different wt% of TiO_2_ with chitosan were prepared (as given in experimental part) to investigate the role of the addition of TiO_2_ with chitosan in the uptake of MNPs, therefore these nanocomposites of different wt% of TiO_2_ was further dip in solution containing different metal ions. The metal ions was templated on the surface of CS-TiO_2_ composite fibers due to their interaction with amine and hydroxyl groups present in the chitosan chains and the TiO_2_ increase the surface area for metal ions adsorption as previously reported^63,64^. Besides, it is reported in literature that NaBH_4_ has standard reduction potential of −1.33 V in aqueous system^65^. Which mean, NaBH_4_ is able to reduce any metal salt with reduction potential higher than −1.33 V. The selected metals for this study were copper, nickel, cobalt and silver because their reduction potentials are 0.342 V, −0.230 V, −0.280 V and 0.799 V, respectively, which favoring the reduction metal ions to zero-valent MNPs by NaBH_4_. Therefore, the nanocomposite fibers of CS-TiO_2_ loaded with metal ions were treated with freshly prepared NaBH_4_ aqueous solution. The metal ions templated on the surface of CS-TiO_2_ nanocomposite fibers was reduced to zero-valent MNPs by NaBH_4_ as discussed earlier.

### Characterization of CS-TiO_2_ nanocomposite fiber and SMNPs

#### FTIR analysis

**Figure 2:**
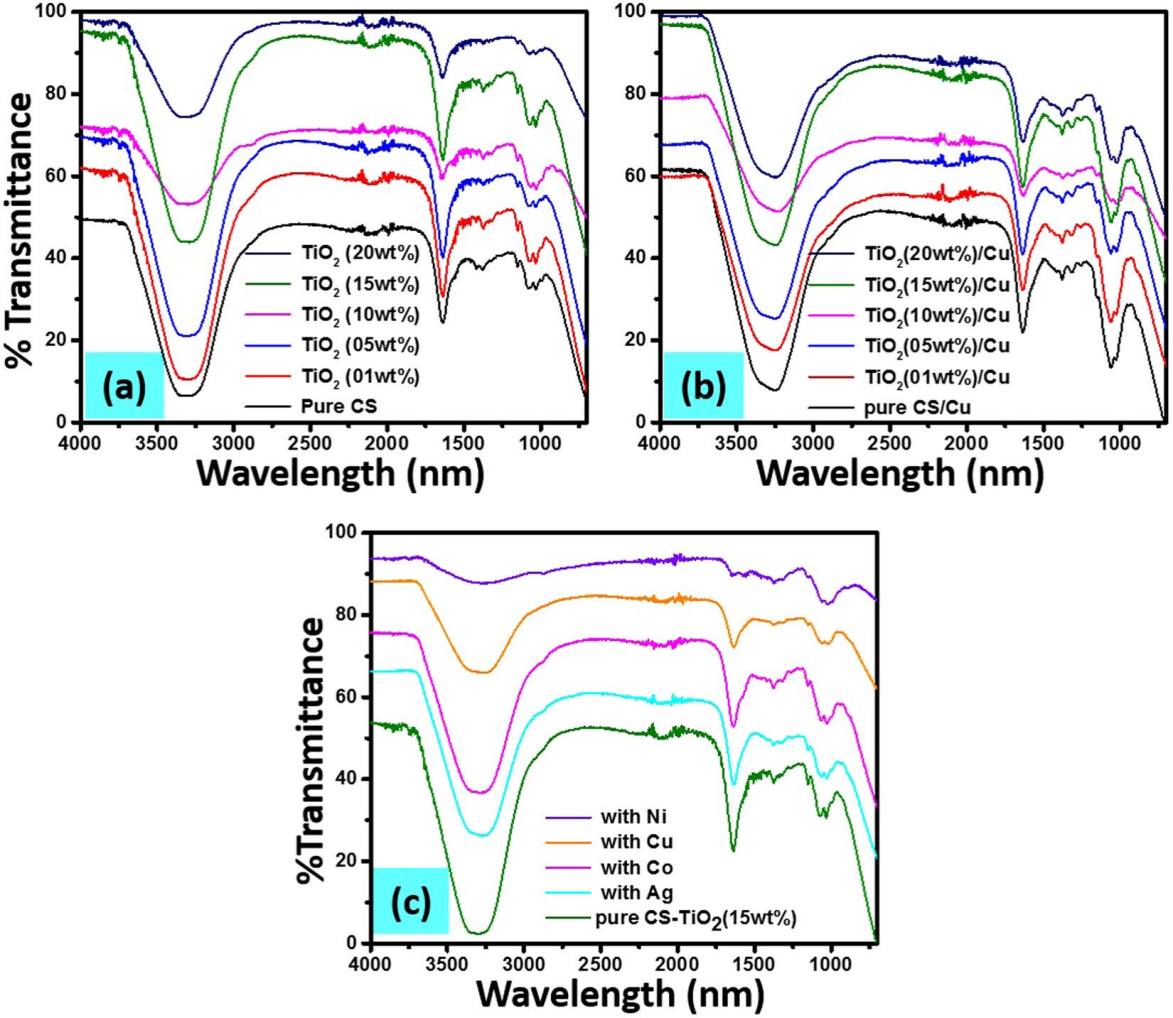
FT-IR spectra of pure chitosan and loaded with different wt% (1, 5, 10, 15, 20 wt%) of TiO_2_ (**a**) and their respective zero-valent Cu nanoparticles loaded nanocomposites (**b**), pure CS-TiO_2_ (15 wt%) nanocomposite and templated with different MNPs (**c**).

The FTIR spectra of pure chitosan and synthesized CS-TiO_2_ nanocomposite fiber with different wt% of TiO_2_ is presented in Fig. 2a, and their respective spectra of templated Cu nanoparticles can be observed in Fig. 2b. Similarly, the FT-IR spectra of the pure CS-TiO_2_ (15 wt% of TiO_2_) nanofiber and loaded with different MNPs is shown in Fig. 2c. In all spectra, the broad band can be observed from 3200–3400 cm^−1^, which was assigned to the presence of N-H and O-H groups in the chitosan chain^63^. The sharp band at 1640 cm^−1^ correspond to N-H bending in amide group of chitosan. The characteristic bands of any polysaccharide such chitosan can be observed at 1030, 1085, and 1375 cm^−1^ was due to O-H bending vibration, C-O-C asymmetrical stretching vibration and C-N stretching vibration respectively^66,67^. These characteristic band of chitosan can be easily observed in all spectra of nanocomposite fibers of chitosan with different wt% of TiO_2_ and templated with various MNPs (Fig. 2a–c). There is no any clear difference in the spectra of pure chitosan and the nanocomposite fiber of CS-TiO_2_ with different wt% of TiO_2_ as well as the metal loaded nanocomposite fiber, which suggest that the addition of TiO_2_ (no matter upto 20 wt%) and loading of MNPs didn’t change the chemical composition of chitosan. Therefore, it was suggested from the FT-IR study that TiO_2_ was physically present in the chitosan fiber without affecting its chemical structure by making or breaking of the chemical bonds.

#### XRD analysis

**Figure 3:**
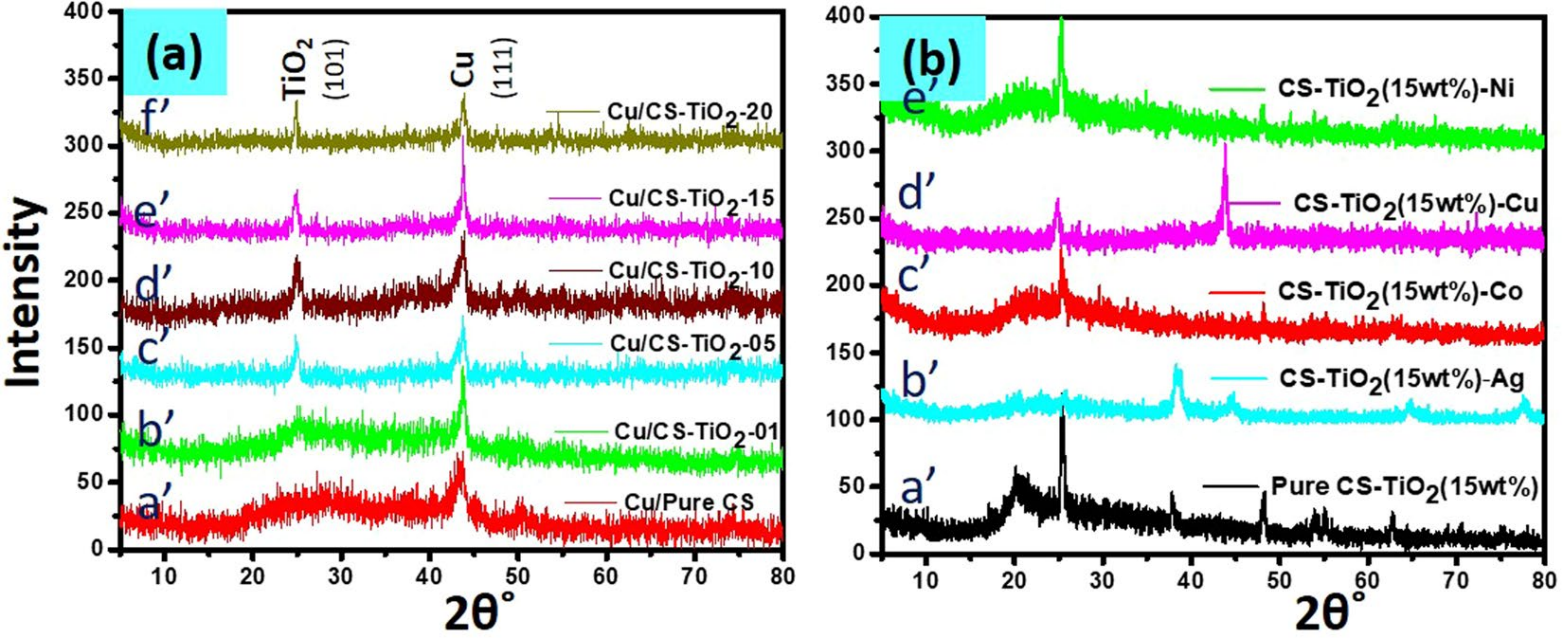
XRD pattern of nanocomposite fibers templated metal nanoparticles.

The structure and crystalline phase of the synthesized SMNPs were analyzed using XRD. Figure 3a,b exhibits the XRD patterns of templated MNPs on pure CS and with different wt% of TiO_2_ nanocomposites. A broad diffraction between 20° and 30° can be observed in the XRD pattern of pure CS fibers, which suggests the amorphous nature of the fibers (Fig. 3a-a’). Previous studies also reported the broad peak at 2θ value less than 30° for the amorphous nature of chitosan^68^. The peak at 25.3° was absent in the diffraction spectra of pure CS while it can be observed in the patterns of nanocomposites of CS-TiO_2_ (diff. wt%) is due to TiO_2_, which enhance with increasing the wt% of TiO_2_, suggesting successful nanocomposites preparation of CS with different wt% of TiO_2_. The peak at 25.3° shows the appearance of crystalline TiO_2_ in anatase phase having (101) facets which are in agreement with JCPDS card no 21–1272. It can be observed from the diffraction peak related with the TiO_2_ in the diffraction patterns, presenting higher intensities when the amount of TiO_2_ nanomaterial is higher in the nanocomposite, confirming the anatase crystalline form of TiO_2_ nanoparticles in the nanocomposites with increasing intensity in the region of TiO_2_ corresponding to the quantity of TiO_2_ added^47^.

In present study, different MNPs (Cu, Co, Ag and Ni) were grown on these prepared CS-TiO_2_ nanocomposites fibers. The synthesis of MNPs templated on nanocomposite was confirmed by using X-ray powder diffractogram. The catalytic efficiency of Cu-NPs templated with CS-TiO_2_ nanocomposite was greater as compare to other MNPs. Therefore, templated Cu-NPs on CS and CS-TiO_2_ (diff. wt% of TiO_2_) nanocomposite can observed in Fig. 3a. The main peak at 2θ = 43.3° can be observe in all diffraction patterns (pure CS and CS coated with TiO_2_), which exhibits the presence of Cu-NPs. The peak at 2θ = 43° correspond to the (111) crystal plane of Cu-NPs. The observed peak of face center cubic (FCC) Cu-NPs at 43° was consistent with previous reports and JCPDS library (JCPDS 04-0836), which suggest the successful synthesis of Cu-NPs on nanocomposite fiber^69^. In one of the previous report, Cu-NPs were synthesized by treating CuCl_2_ salt with NaBH_4_ in the presence of cobalt tetraamino phthalocyanine as a stabilizer. The same XRD pattern was observed for resulting Cu-NPs having peaks at 43° for FCC before exposing to air. On exposing to air for 24 hours, the broader peaks at 36.8°, 60.9° was observed due to formation of CuO-NPs and a very weak peak were observed for Cu-NPs^70^. Thus, FCC crystals of Cu-NPs were successfully synthesized on all the surfaces. The crystal size was analyzed by fityk software and found to be less than 1 nm size for the Cu-NPs templated on these various compositions of nanocomposite. We investigated the catalytic efficiencies of all composition of nanocomposite fiber and found that 15 wt% of TiO_2_ nanocomposite have greater efficiency than the other nanocomposites (shown in 3.3 portion of results), therefore, pattern of comparing various MNPs templated on CS-TiO_2_-15 are shown in Fig. 3b.

Figure 3b(a’-f’) shows the pattern of neat CS-TiO_2_-15 and loaded with different MNPs. The pure CS-TiO_2_-15 has a broad band around 20° is due to the amorphous nature of chitosan and the other peaks at 2θ = 25.37, 38.04, 48.17, 55.07, 62.6 and 75.47° corresponding to the (101), (004), (200), (211), (002) and (215) planes of TiO_2_ and in agreement with JCPDS, 21- 1272 and with previous reports^71,72^. The corresponding CS-TiO_2_-15 loaded with different MNPs were shown in Fig. 3bb’-e’. Figure 3bb’ exhibits the pattern of the nanocomposite templated with Ag-NPs, which shows the peaks at 2θ = 38.60, 44.51, 64.63 and 77.72° which corresponds to the (111), (200), (220) and (311) planes of Ag and were in good agreement with our previous and other reported data^73,74^. In this pattern the peak for TiO_2_ cannot be clearly observed due to the high intensity of the Ag-NPs which are embedded on the surface. In case of loaded Co-NPs (Fig. 3bc’), the strong peak was observed at 25.37° and a small peak at 48.17°, which exhibits the presence of TiO_2_. According to JCDPS card no 15-0806, Co has sharpest peak at 43.8° corresponding to (111) Co crystal plane, which were not observed in the present study, which might be due to the low contents of these NPs on the nanocomposite surface. Since no other peaks were observed for cobalt oxide and hydroxide, which suggest the successful formation of Co-NPs. In order to investigate the presence of Cu-NPs templated on the CS-TiO_2_-15 nanocomposite fibers, the pattern was recorded as shown in Fig. 2bd’. In this pattern, the main peak can be observed at 2θ = 43° and other small peak at 24.81°, corresponding to (111) crystal plane of copper and (101) plane of TiO_2_ respectively. The XRD pattern of the nanocomposite templated with Ni-NPs can be observed in Fig. 3be’. Ni-NPs has a sharpest peak at 2θ = 45° for (111) reflection, which was absent in our present study. However, Ni-NPs has broad halo peak at 2θ = 45° which was not present in the XRD pattern of pure CS-TiO_2_-15 nanocomposites. The other peaks related to Ni-NPs were not observed due to low content of the Ni-NPs over composite fibers. Our results are in good agreement with some of the previously reported articles. In one report Ni-NPs was prepared by choosing the treatment of NiCl_2_ with NaBH_4_, using chitosan coated filter-paper as supporting material^41^. They observed the similar XRD pattern, having broad halo peak at 2θ = 45° for Ni-NPs. Similarly, in another report, Ni-NPs were synthesized by treating NiCl_2_ with NaBH_4_, without using any supported material^75^. The similar XRD pattern was also observed by them, where for Ni nanomaterials the only broad peak at 2θ = 45° was identified. Since, in the XRD pattern of Ni loaded, no other than the peak for TiO_2_ were observed, which suggested that Ni-NPs were successfully prepared without its conversion into nickel oxide and hydroxide. As MNPs are highly unstable and radially oxidizes into metal oxides. Therefore, the formation of metal oxides with time cannot be ignored. However, observing the XRD patterns of all loaded MNPs didn’t exhibits any peaks for their oxides which confirm the successful synthesis of MNPs on the nanocomposite. Thus, XRD patterns demonstrates the successful synthesis of MNPs on the surface of CS coated with different wt% of TiO_2_

#### FE-SEM analysis

**Figure 4:**
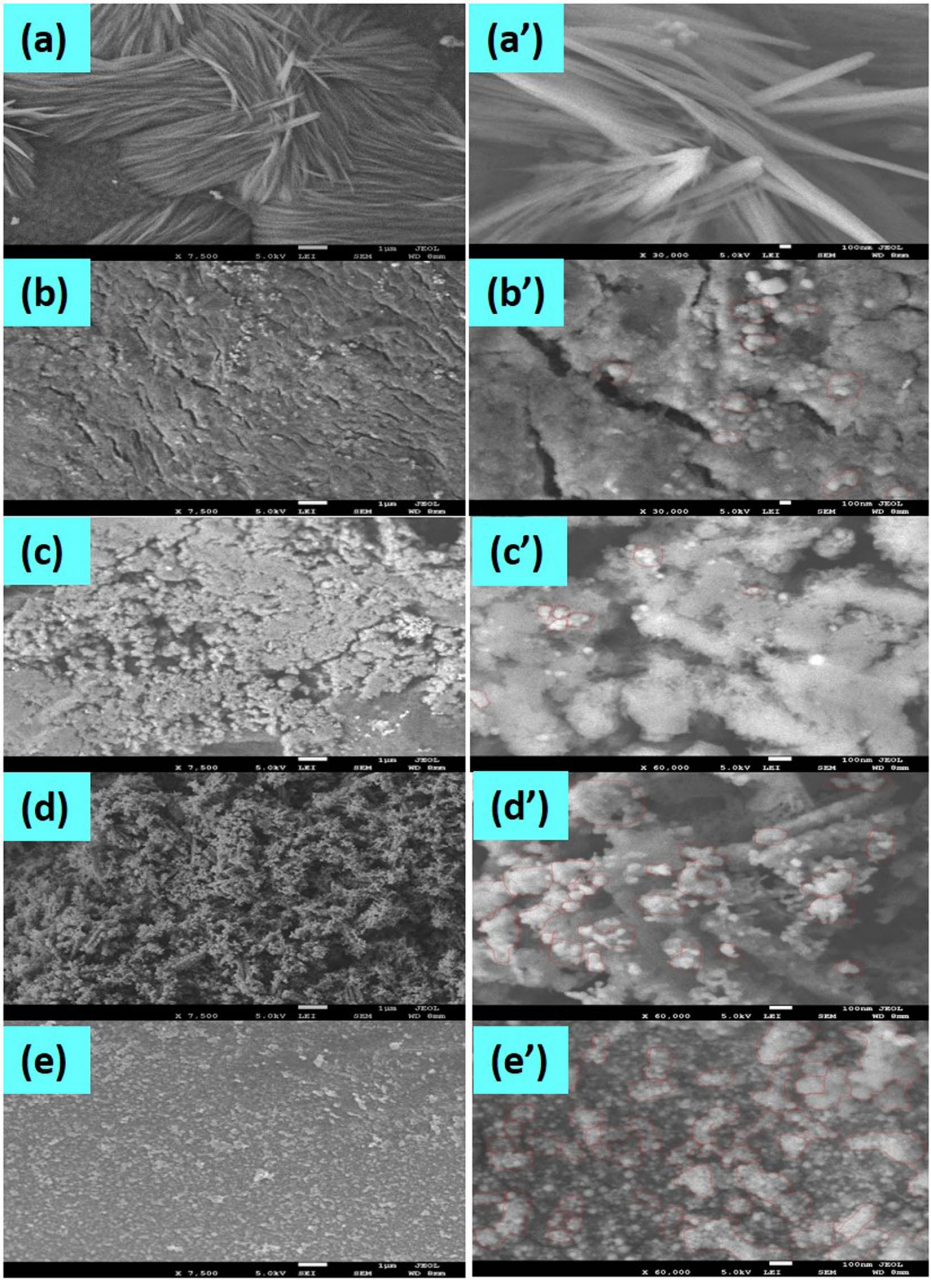
FESEM images of pure CS-TiO_2_ fibers (**a**,**a’**), Cu/CS-TiO_2_-15 (**b**-**b’**), Co/CS-TiO_2_-15 (**c**-**c’**), Ag/CS-TiO_2_-15 (**d**-**d’**) and Ni/CS-TiO_2_-15 (**e**-**e’**).

As we get better catalytic activity of zero-valent MNPs supported on CS-TiO_2_-15 nanocomposite, therefore, the morphologies of the neat CS-TiO_2_-15 nanocomposite and templated with different MNPs were analyzed using FE-SEM as shown in Fig. 4. The low magnification (7,500 × ) images of the pure and metal loaded nanocomposite are represented on left side in Fig. 4a–e, while their respective high magnification (60,000 × ) images on the right side in Fig. 4a’–e’. We can clearly observe the nano-fibrous structure formation from the low and high magnification of pure CS-TiO_2_-15 fibers (Fig. 4a-a’). In the first image of neat CS-TiO_2_-15 fibers no loaded materials/NPs can be observe, which suggest that MNPs were not templated on that fibers. In contrast to the first image, in the other images of the nanocomposite loaded with different MNPs (Fig. 4b–e), the small dots/nanoparticles can be easily observed which suggest that MNPs were successfully templated on the nanocomposite. These images also suggested that MNPs were well dispersed on the surface of CS-TiO_2_ nano-fibrous structure. These MNPs covered most of the surface of CS-TiO_2_ nanocomposite due to the presence of N-H_2_ and O-H groups in the chitosan chain and the presence of TiO_2_ which increase the surface area for metal ions uptake^63,64^. The average sizes of MNPs were determined by using Image J software, which were 28.73 nm 26.66 nm, 32.80 nm, 26.51 nm for Cu, Co, Ag and Ni, respectively, templated on CS-TiO_2_-15 fibers. The red circles on the images exhibited the aggregation occur in the nanoparticles, more aggregation can be observed in case of Ag and Ni nanoparticles templated on the nanocomposite which might be the possible reason for their low catalytic efficiency. It was not possible to elucidate from SEM analysis that TiO_2_ and MNPs were present in templated CS-TiO_2_ nanocomposite because its only reveals the surface morphology of the samples. Therefore, EDS was used to confirm the presence of MNPs and TiO_2_ in the chitosan host.

#### EDS analysis

**Figure 5:**
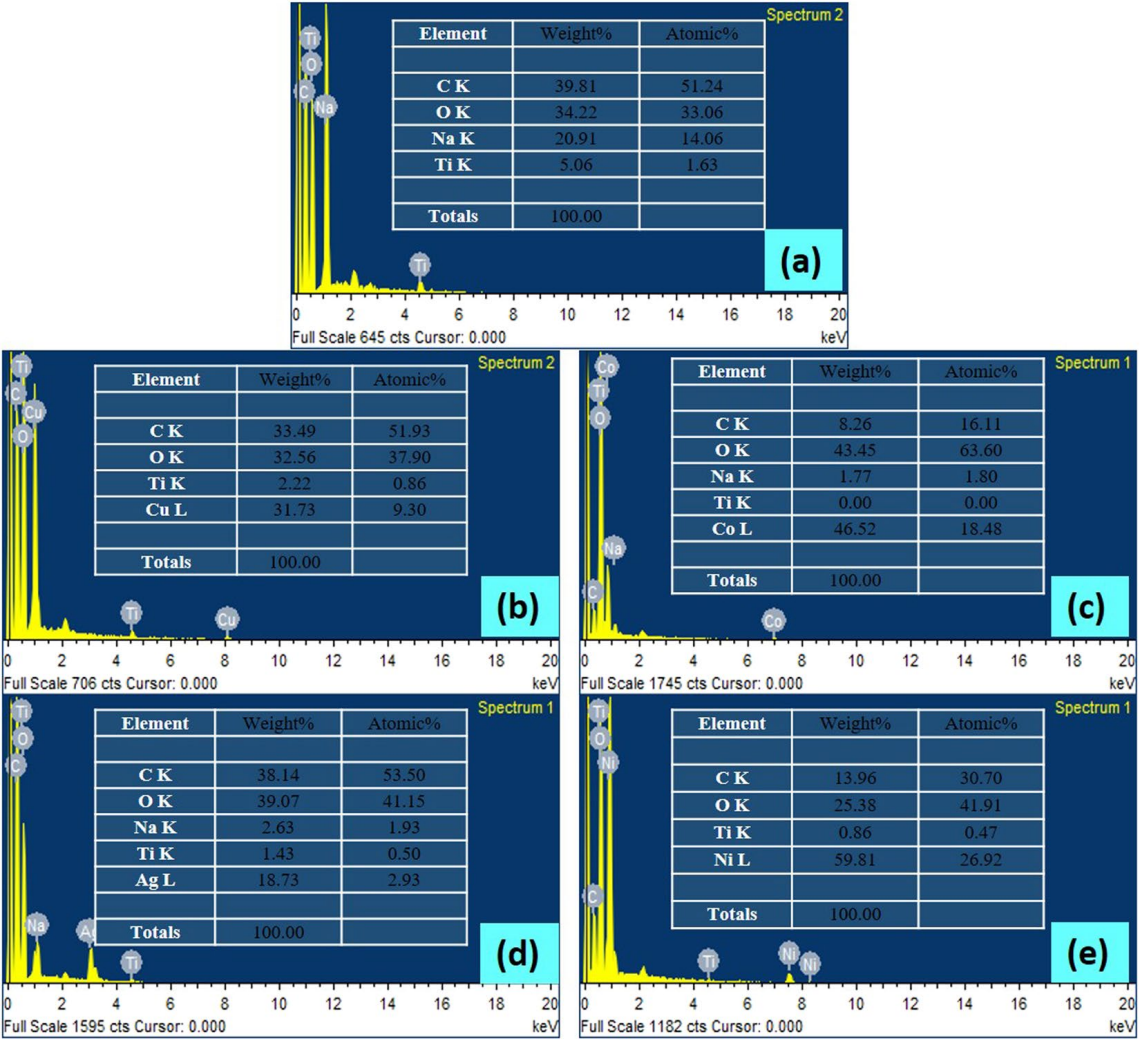
EDS of pure CS-TiO_2_-15 fibers (**a**), Cu/CS-TiO_2_-15 (**b**), Co/CS-TiO_2_-15 (**c**), Ag/CS-TiO_2_-15 (**d**) and Ni/CS-TiO_2_-15 (**e**).

The successful MNPs synthesis in CS-TiO_2_-15 nanofibers was also confirmed by EDS analysis, where elemental Cu, Co, Ag and Ni can be clearly observed in the sample composition (Fig. 5b–e). The presence of titanium can also be observed in the sample composition of the neat and MNPs templated CS-TiO_2_-15, which suggest the successful synthesis of the nanocomposite of CS with TiO_2_. Other elements of carbon and oxygen observed in the EDS spectrum were mostly due to the various functional groups present in the CS. Na was also detected at minor concentration due to the fiber formation of the nanocomposite in aqueous NaOH solution.

### Catalytic Activity

#### MO dye reduction

MO is widely used as a pH indicator in industries and research institutes whose chemical structure comprise an azo group. The removal of MO dye from the environment gain great interest because it causes several health and environmental related problems^76,77^. The catalytic reduction of organic dyes by NaBH_4_ in the existence of catalyst is widely applied to evaluate the catalytic properties of various catalysts. This reduction reaction of MO is thermodynamically favorable but unfavorable kinetically^4,78^. MO imparts an orange-red color to the aqueous solution. A strong absorption band at 464 nm and another band at 280 nm were recorded for the aqueous solution of MO using UV-visible spectrophotometer. The absorption band at λ_max_ = 464 nm was due to the presence of -N = N- functional group in the chemical structure of MO. The reduction of MO by NaBH_4_ in the absence of suitable catalyst is kinetically slow and take several hours as previously reported^79^. Such studies suggest that this reaction is kinetically unfavorable by using only NaBH_4_ without the use of catalyst. Herein, we used of different MNPs (Ag, Co, Cu and Ni) templated on CS-TiO_2_-15 as a catalyst to investigate the catalytic reduction of MO by NaBH_4_.

**Figure 6:**
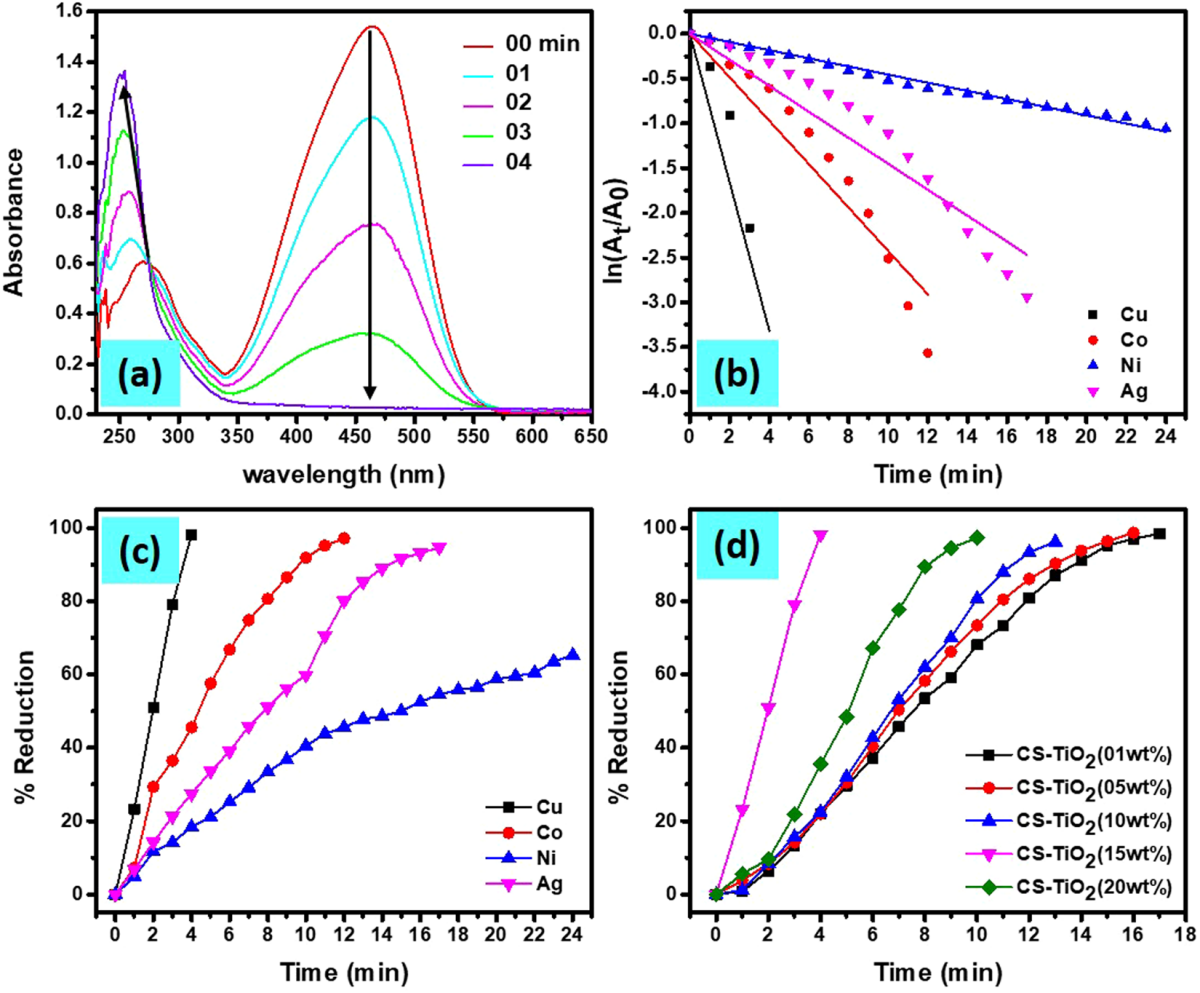
UV-vis spectra of MO dye reduction as a function of time in the presence of Cu/CS-TiO_2_ (**a**), Ln (A_t_/A_0_) vs time of different MNPs templated on CS-TiO_2_-15 (**b**) Percent reduction of MO with time by different MNPs templated on CS-TiO_2_-15 (**c**) and by Cu-NPs templated on different wt% of TiO_2_ in CS-TiO_2_ (**d**) nanocomposite

UV-visible spectra of the solution in UV cuvette containing 3 mL of 0.01 mM MO and 0.5 mL of 0.1 M NaBH_4_ in the presence of 25 mg of prepared catalysts were monitored and recorded after every minute. Figure SI-1a–d and SI-2a–e exhibited the UV-vis spectra of MO reduction by NaBH_4_ as a function of time in the presence of different MNPs templated on CS-TiO_2_-15 and Cu-NPs loaded on different wt% of TiO_2_ in CS-TiO_2_ nanocomposite, respectively. The regular decrease in intensity at λ_max_ = 464 nm and increase and shift of absorption band from 270 nm towards 250 nm with time can be observed in all spectra. These results suggest that the -N = N- site of MO was reduced by NaBH_4_ in the presence of catalyst and produces amino compounds of low molecular weight. Therefore, the appearance of the new peak at 250 nm (Fig. 6a), during the MO reduction was considered as the formation of -NH_2_ compounds^79^. The *ln (A*_*t*_*/A*_0_) and percent reduction of MO as a function of time were calculated from the UV-visible spectra by using the following equations:

5 $$Ln({A}_{t}/{A}_{0})={k}_{app}xt$$

where A_0_ was the initial absorbance of MO at zero time at λ_max_ = 464 nm, while A_t_ is the apparent absorbance at time ‘t’ at the same wavelength. The recorded absorbance after every minute at λ_max_ = 464 nm for MO were drawn as *(A*_*t*_*/A*_0_) versus time to investigate the catalytic activity of MNPs and wt% of TiO_2_ in nanocomposite (Figs SI-1e and 2f). The ratio of *A*_*t*_/*A*_0_ as a function of time in the presence Cu/CS-TiO_2_-15, decreased abruptly (within 4 min) as compared to other MNPs and weight percent of TiO_2_, which suggest that Cu-NPs loaded on CS-TiO_2_-15 exhibits good catalytic activity for MO reduction as compared to other MNPs and composite composition. The gradual desertion of the peak at 464 nm with the addition of Cu/CS-TiO_2_-15 nanocomposite was due to reduction of MO solution, which was completed in very short time of 4 min (Fig. 6a). The straight line can be observed by plotting ln *(A*_*t*_*/A*_0_) versus time (Fig. 6b), which suggest that this reaction followed *pseudo first order* kinetic (Equ. 5). Figure 6c and d, exhibits the percent reduction of MO calculated by Equ. 4, which clearly demonstrate that Cu-NPs templated on CS-TiO_2_-15 take shorter time for the complete reduction of MO as compared to the other MNPs and weight percentage of TiO_2_ nanocomposite. The regular decrease in time was observed by increasing the amount of TiO_2_ in the nanocomposite, which suggest that TiO_2_ play its role in the uptake of metal ions. This was ascribed to the enhance in the active sites for the uptake of MNPs which were responsible for MO dye reduction^80^. Moreover, this catalytic reaction was taking place in the UV-cuvette, where UV-light passed through the sample in every reading, therefore higher TiO_2_ content might absorb more UV-light and thus speed up the reaction^81^. However, the reduction efficiency decreased pointedly with higher content of TiO_2_ (20 wt%), due to agglomeration of TiO_2_ particles in the polymer matrix which reduced the surface area responsible for the uptake of MNPs and UV-light absorption capacity^82^. The detail study of MO dye reduction by NaBH_4_ was carried in the presence of Cu-NPs templated on CS-TiO_2_ nanocomposite havig15wt% of TiO_2_ were used as a catalyst, which is represented as Cu/CS-TiO_2_-15.

#### Effect of light on MO dye reduction

**Figure 7:**
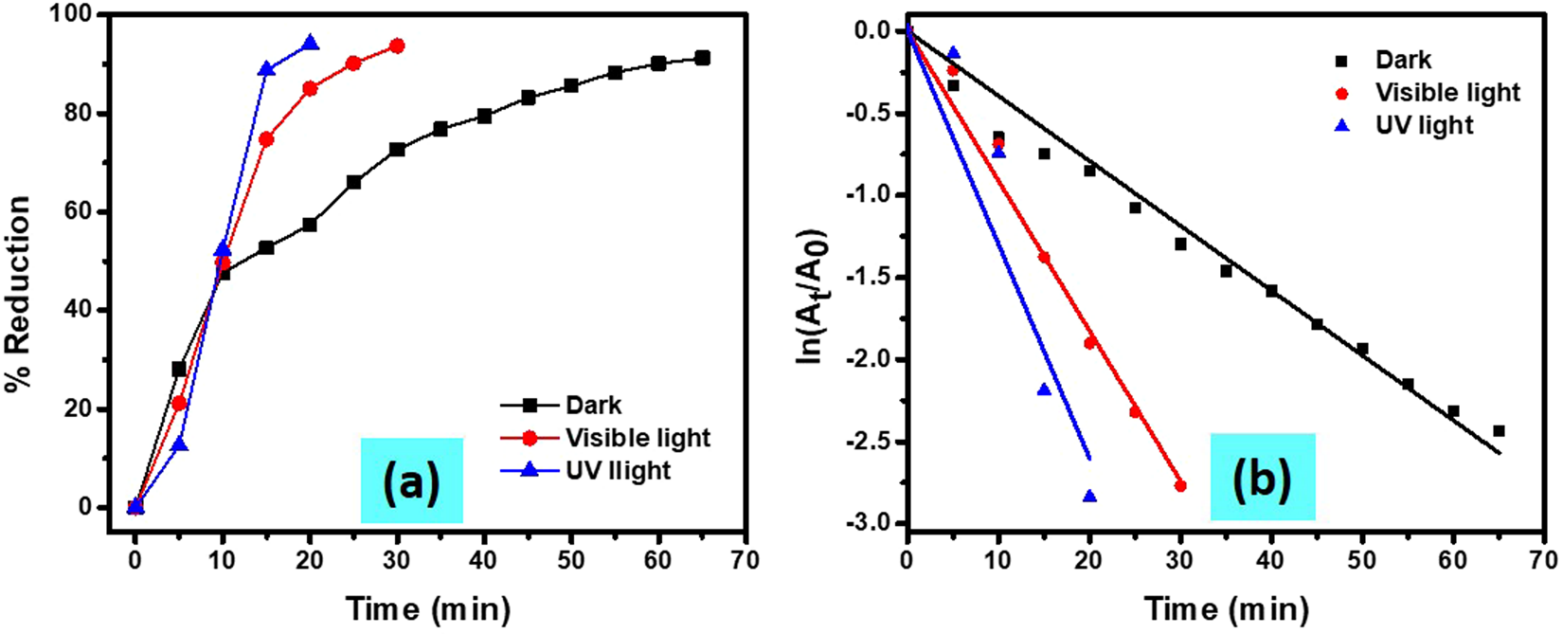
Percent reduction of MO by NaBH_4_ as a function of time by using catalyst (Cu/CS-TiO_2_-15) in presence and absence of visible light and UV-radiation (**a**). And their respective Ln (A_t_/A_0_) values vs time (**b**).

Since, the percent reduction of MO dye was enhancing with increasing wt% of TiO_2_ in the nanocomposite, therefore the efficiency of Cu/CS-TiO_2_ was investigated in dark as well as by irradiating the reaction vessel with UV lamp and visible light. The catalytic reduction of MO dye as a function of time by NaBH_4_ and Cu/CS-TiO_2_ in the presence and absence of UV and visible light was monitored and recorded as shown in Fig. SI-3. In the absence of light source, Σ 90% of dye was reduced by NaBH_4_ within the contact time of 65 min (Fig. 7a). However, in presence of UV and visible light this much of reduction was obtained only in 15 min and 25 min, respectively. The efficiency for dye reduction were increased 1/4.33 time in presence of UV-irradiation and 1/2.60 time in case of visible light. The remarkable dye reduction in presence of UV-irradiation clearly explain the role of TiO_2_ distributed over the chitosan surface as photocatalyst. The order of reaction in presence and absence of UV and visible light was determined by using *pseudo first order* kinetic equation as shown in Equ. 5. A straight line was obtained having adj-R^2^ value more than 99% was obtained for all reaction matrix as manifested in Fig. 7b, which confirm that these reactions followed *pseudo first order* kinetics because NaBH_4_ was used in excess as compared to MO concentration.

#### Effect of catalyst amount

**Figure 8:**
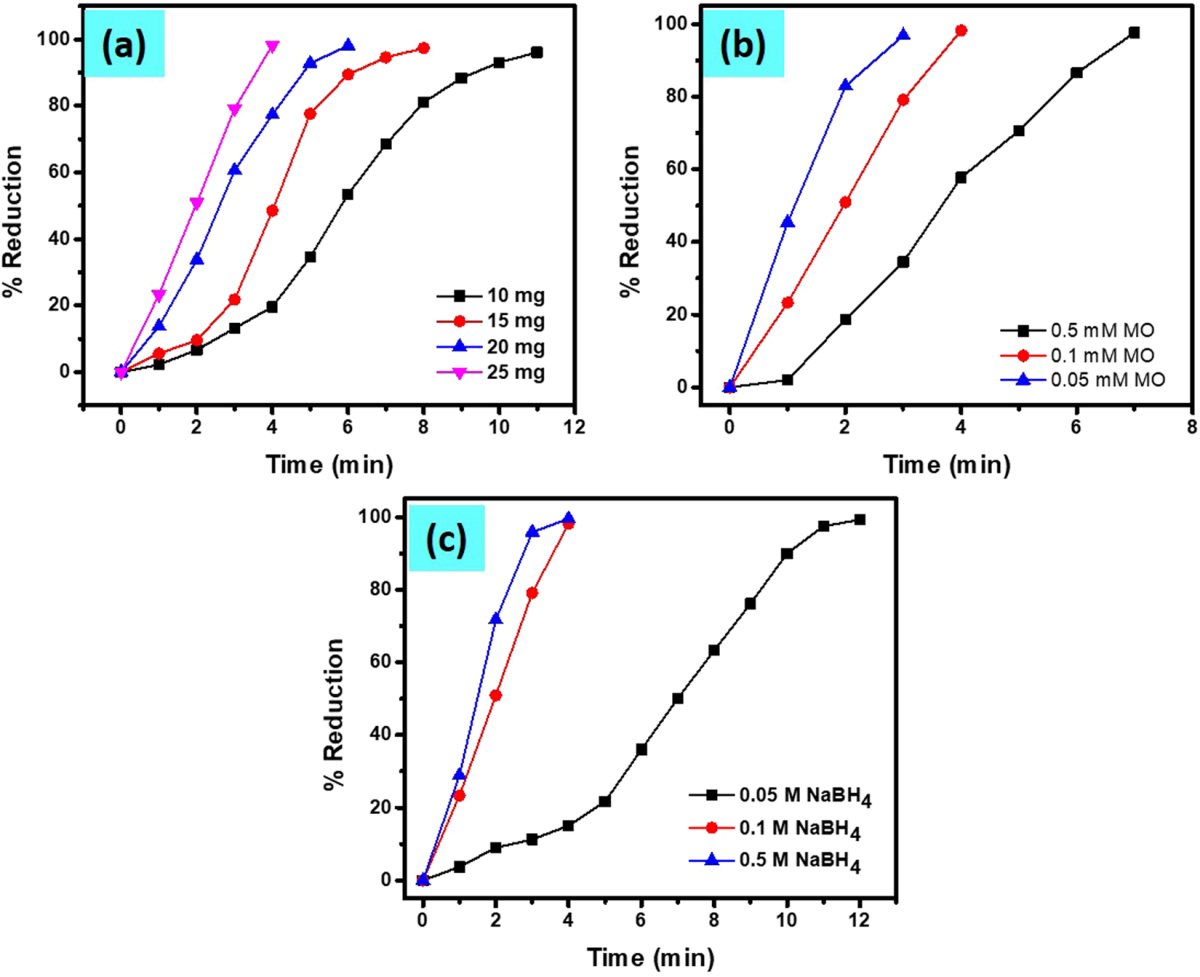
Percent reduction of MO dye by NaBH_4_ in the presence of different catalyst amount (Cu/CS-TiO_2_-15) (**a**), different initial concentration of MO (**b**) and different initial concentration of NaBH_4_ (**c**).

Different amount of the Cu/CS-TiO_2_-15 nanocomposite fibers were used to investigate the catalytic reduction of MO by NaBH_4_. Figure SI-4a–d shows the UV-vis spectral graphs as function of time for MO dye solution, where 10 mg, 15 mg, 20 mg and 25 mg Cu/CS-TiO_2_-15 nanocomposite fibers were used as catalysts, respectively. The only difference between these plots was that the MO reduction was completed within less time for the catalytic fiber having greater amount (25 mg). Obviously, the number of Cu-NPs was greater in 25 mg than in the rest of the fibers, which led to quicker completion of the reduction reaction. (Fig. SI-4e) shows the ratio of *A*_*t*_*/A*_0_ (absorbance at λ_max_ 464 nm) of MO with respect to time in the presence of different amount of SMNPs as a catalyst. The time taken for the reduction of MO was observed to decreased with increasing the amount of the catalyst. The minimum time taken for complete reduction of MO dye by 25 mg of nanocomposite can be observed from Fig. 8a.

#### Effect of NaBH_4_

Besides the effect of the catalyst amount on MO reduction reaction, the effect of the initial concentration of MO and NaBH_4_ was also investigated using a constant amount of catalyst (25 mg Cu/CS-TiO_2_-15). Figure SI-5a–c shows the UV-vis spectra of the catalytic reduction of different initial concentration of MO dye by the constant amount of NaBH_4_ (0.5 mL of 0.1 M) and Cu/CS-TiO_2_-15 (25 mg) for each reaction system. Three concentrations 0.5 mM, 0.1 mM and 0.05 mM of MO dye were tested keeping other parameters constant (*i.e* 3 mL of each concentration of MO plus 0.5 mL of 0.1 M NaBH_4_ and 25 mg of the nanocomposite). The ratio of A_t_/A_0_ for different initial concentration was plotted as a function of time, the gradual increase in time can be observed for high concentration of the dye (Fig. SI-5d). Moreover, the percent reduction of MO was calculated using Eq. 4 and exhibited in Fig. 8b. A general trend of decreasing rate of reduction was found with increasing MO concentration. Moreover, three different initial concentration (0.5 M, 0.1 M and 0.05 M), NaBH_4_ were tested keeping other parameters constant, like same amount and concentration of MO dye (3 mL of 0.05 mM) and catalyst (25 mg Cu/CS-TiO_2_-15). The data obtained are presented in Fig. SI-6a–d. The percent reduction calculated by using Eq. 4 is shown in Fig. 8c. The decrease in time with increasing the concentration of NaBH_4_ was observed. However, this affect was not observed by increasing concentration of only NaBH_4_ without using catalyst, which suggest that in high concentration of NaBH_4_, high number of BH_4_^−^ ions were available which diffused into the MNPs present on the surface of the CS-TiO_2_-15. Based on the results obtained for MO reduction as described in above text, the mechanism for catalytic reduction of MO over Cu/CS-TiO_2_-15 is given. The MO and BH_4_^−^ are first get adsorbed into the catalyst and as a consequence of this, the adsorbed electrons and hydrogen species on the surface of catalyst are transferred to MO, thus resulting in the reduction of -N = N- group to the -NH_2_ group.

#### Catalytic reductions of CR, MB and AO

We also investigated the catalytic activity of Cu/CS-TiO_2_-15 in the catalytic reduction of CR, MB and AO dyes. CR is another notorious dye responsible for water pollution. CR is a contaminant that is considered to causes carcinogenicity and great harm to marine environments. CR is benzidine base anionic azo dye is considered as human carcinogen. The thermal, optical and physiochemical stability of CR is due to the presence of aromatic structure in its chemical composition^83^. Two azo groups are present in the chemical structure of CR. In literature, it is reported that azo dyes reduction leads to the breaking of the -N = N- bond which results to the formation of aromatic amines^84,85^. As every molecule of CR is composed of two -N = N- groups, therefore a slower reduction reaction was expected. Recently, different studies were carried on MB and AO dyes removal which also causes environmental pollution^86,87^. Therefore, removal of such toxic dyes from wastewater is very essential before it is discharged into aquatic environment.

**Figure 9:**
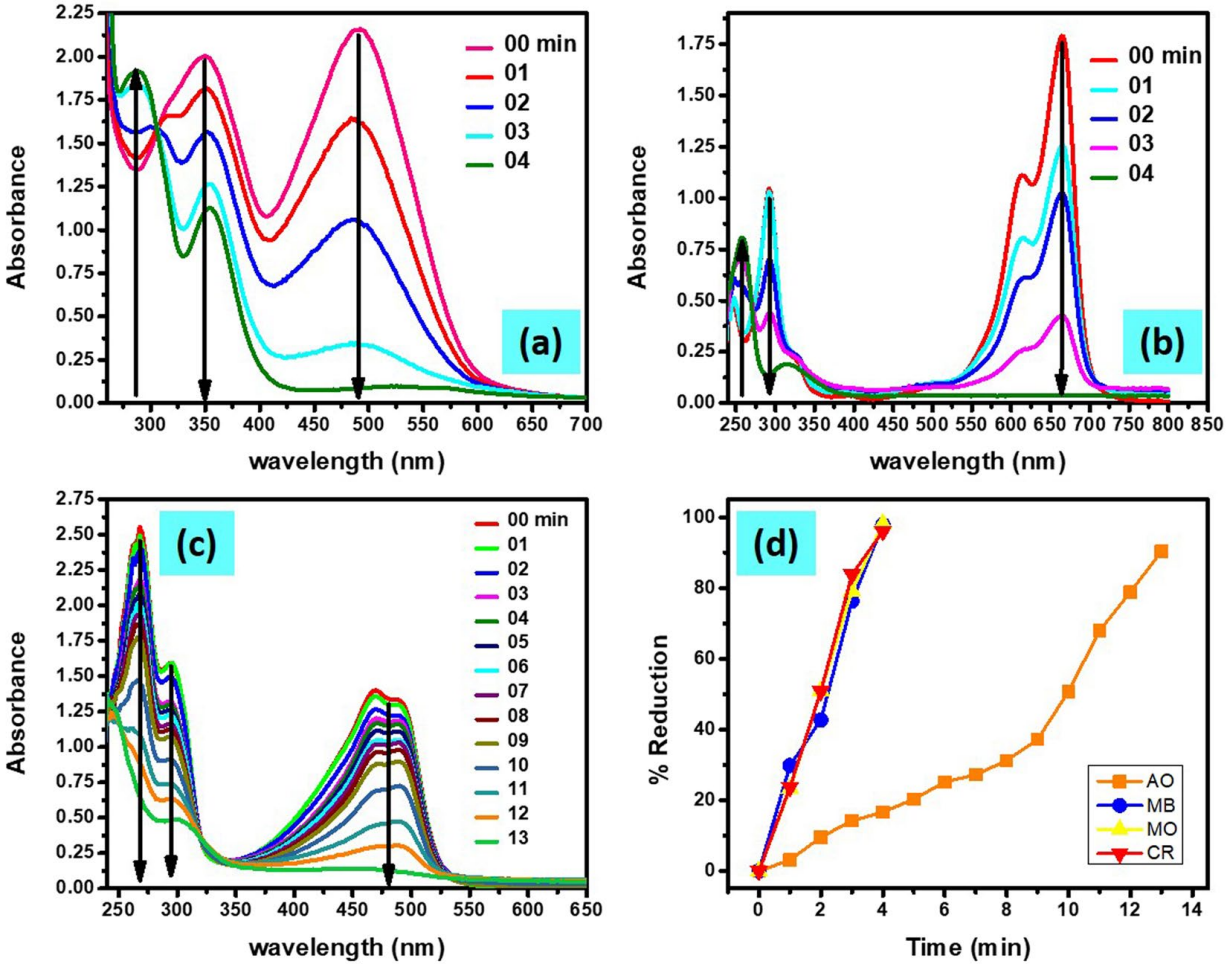
UV-visible spectra of CR (**a**), MB (**b**), and AO (**c**) solutions containing Cu/CS-TiO_2_-15 and percent reduction of MO, CR, MB and AO as a function of time (**d**).

Just like previous experiments, the UV-visible spectra of the solution of CR, MB and AO separately with NaBH_4_ in a cuvette were monitored and recorded with a constant and regular interval of time in the presence of Cu/CS-TiO_2_-15 nanocomposite. Figure 9a exhibits the UV-visible spectra of the CR solution containing Cu/CS-TiO_2_-15. Two peaks can be observed at 350 nm and 492 nm due to the presence of CR in the solution. A clear decline can be observed with time in the absorbance of both peaks. Besides, a new peak at 290 nm was evolved with time. The observed changes in the UV-visible spectra of CR solution suggest that in the presence of Cu/CS-TiO_2_-15 nanocomposite the original molecules of CR converted to smaller molecules by NaBH_4_. Similarly, Fig. 9b shows the UV-vis spectra of the MB solution measured at constant interval of 1 min between two runs in the presence of Cu/CS-TiO_2_-15 as a catalyst for the reduction of MB by NaBH_4_. In case of MB, also two absorbance peaks were observed at wavelength 292 nm and 664 nm. The regular decrease in both peaks was observed as a function of time. Moreover, a slight blue shift and the evolution of the new peak at 258 nm was also observed, which suggest the successful reduction of MB by NaBH_4_ and formation of amines molecules. In addition to CR and MB, the UV-vis spectra of AO were also recorded by applying the same condition and manifested in Fig. 9c. Two peaks at 268 nm and 294 nm, and one broad absorbance around 480 nm were observed for AO solution. The regular decline in all the absorption peaks can be seen with time, thus AO was also successfully reduced by NaBH_4_. Figure 9d demonstrates the percent reduction of MO, CR, MB and AO as a function of time. The catalytic reduction of MO, CR and MB was 97.9%, 98.2% and 96.0% achieved in the presence of Cu/CS-TiO_2_ within time of 4 min, while in case of AO more than 90% was obtained in 13 min.

#### Nitrophenol transformation to aminophenol

The catalytic transformation of nitrophenols (NP) to aminophenol’s (AP) has been extensively explore from the last decade, because it may be a greener and efficient method for the synthesis of AP^53,88^. AP is an efficient raw material and intermediate for the synthesis of several medicinal and cosmetic products^89^. Therefore, the transformation of NP to AP is a reasonable way to convert toxins to greener and renewable resource. Beside, AP is comparatively less toxic and can be easily removed and mineralized than nitroaromatic compounds^90,91^.

**Figure 10:**
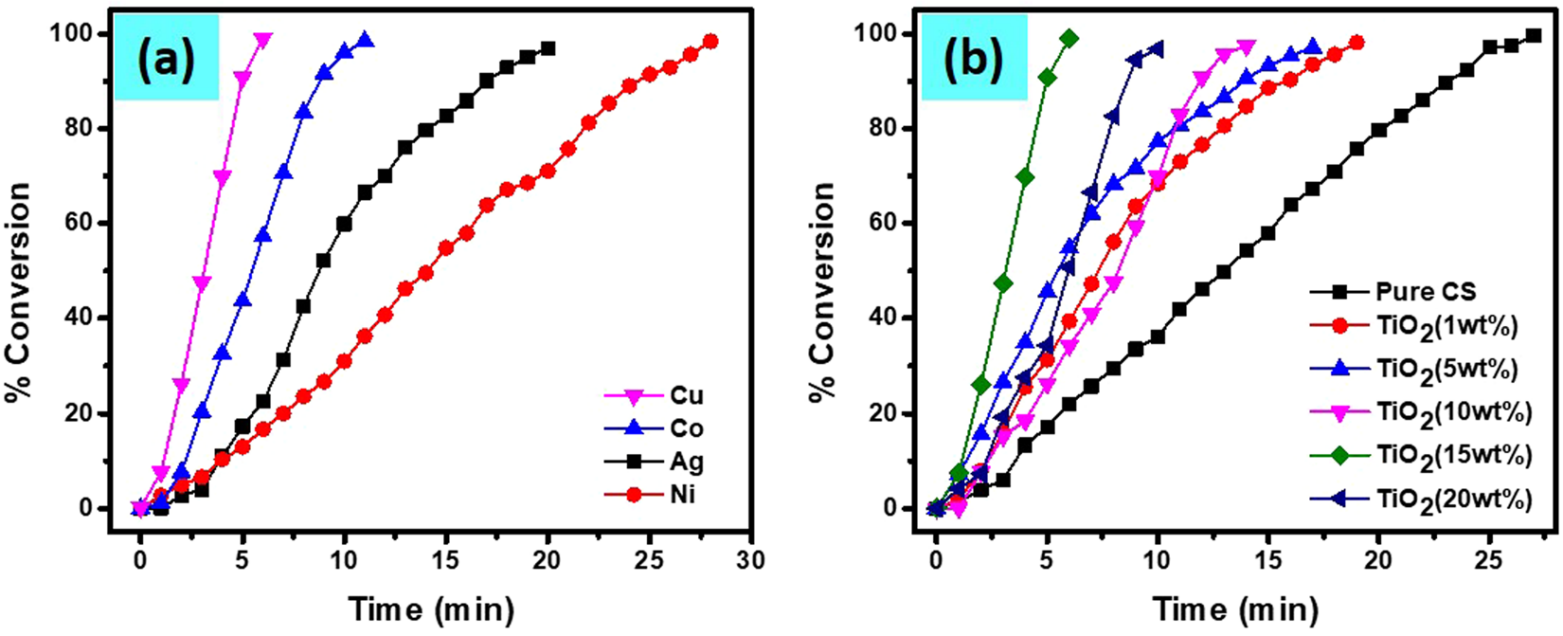
Percent conversion of 4-NP to 4-AP in the presence of catalyst, CS-TiO_2_-15 templated with Cu, Co, Ag and Ni NPs (**a**), and in the presence of Cu-NPs loaded on pure CS and added TiO_2_ of 1 wt%, 5 wt%, 10 wt%, 15 wt% and 20 wt% nanocomposite.

The UV-visible spectra of 4-NP before and after the addition of NaBH_4_ is shown in Fig. SI-7a. The red shift of λ_max_ from 317 nm to 400 nm was observed for the initial solution. Visual investigation suggests that the color of the solution change from light yellow to dark yellow with the addition of NaBH_4_. This color change was due to the formation of 4-nitrophenolate ion as previously reported^18,92^. Even in the excess of NaBH_4,_ no change in this dark yellow color was observed for couple of days, which indicated that 4-NP could not be transformed into 4-AP by only NaBH_4_. The transformation of 4-NP to 4-AP in the presence of NaBH_4_ is thermodynamically favorable because their standard electrode potential is 0.67 V, which is greater than zero^18,41^. However, this transformation reaction of 4-NP to 4-AP in the absence of suitable and efficient catalyst is kinetically unfavorable. Due to this reason, different noble MNPs as a catalyst were tested for the transformation of 4-NP to 4-AP, among which are Pt/C^93^, gold NPs^94,95^ and Fe_3_O_4_@SiO_2_-Ag^96^. Here we tested the catalytic efficiency of different MNPs (Ag, Co, Cu and Ni) templated on nanocomposite of CS-TiO_2_ (diff. wt% of TiO_2_) for the transformation of 4-NP to 4-AP. In the catalytic conversion of 4-NP resulted in a decrease in λ_max_ 400 nm and increase in a new absorption band at 300 nm, which is ascribed to the formation of 4-AP (Fig. SI-7b). Probably, the mechanism of this reaction may involve complicated intermediates located on the surface of the NPs and remains the topic of discussion^88,97^. However, the isosbestic point approximately at wavelength of 320 nm (Fig. SI-7b) exhibits the complete conversion of 4-NP to 4-AP without side reaction^88,98^. UV-vis spectra only show the species in the solution and not the intermediates present on the surface of NPs. Herein, UV-visible spectral data were recorded continuously with a gap of 1-min between two measurements. Figure SI-7b–e shows the UV-vis spectra of CS-TiO_2_ (15 wt%) loaded with different MNPs and Fig. SI-8a–f exhibits the spectra of Cu-NPs templated on pure CS and CS loaded with different wt% of TiO_2_ nanocomposite used as a catalyst for transformation of 4-NP to 4-AP. The gradual decrease in peak intensity at λ_max_ = 400 nm and increased at λ = 300 nm can be observed with passage of time in all the spectra. The decrease of the peak at 400 nm and increase at 300 nm was associated to the transformation of 4-NP to 4-AP with time. The only difference between these plots is the time taken for the completion of the reaction. We get better results for the catalytic conversion of 4-NP in the presence of Cu-NPs templated on CS-TiO_2_-15 as compared to other loaded MNPs on various wt% nanocomposite, which suggest that Cu-NPs are more active towards nitrophenols reduction as compared to other MNPs (Fig. 10a,b). By increasing the amount of TiO_2_ in the nanocomposite enhance the catalytic efficiency unto some limit, which suggest TiO_2_ play a role in the uptake of MNPs (Fig. 10b). Moreover, the catalytic efficiency of nanocomposite of TiO_2_ with 15 wt% of CS in the conversion of 4-NP to 4-AP was greater as compare to other might be due to the photocatalytic properties of TiO_2_ in the presence of UV light. However, the catalytic efficiency was decrease by increasing the amount of TiO_2_ from 15 wt%, which suggest that TiO_2_ mostly covered the active site and surface of CS. Therefore, in case of CS-TiO_2_ (20 wt%), the catalytic efficiency of the nanocomposite was greatly decrease might be due to aggregation or unavailability of active sites for metal ions uptake.

**Figure 11:**
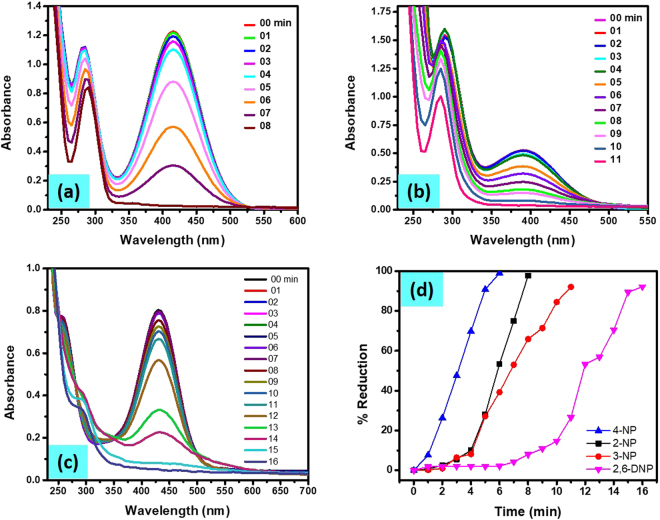
UV-vis spectra of 2-NP (**a**), 3-NP (**b**) and 2,6-DNP (**c**), percent reduction of nitrophenols as function of time (**d**).

We also tested the catalytic efficiency of the Cu/CS-TiO_2_ for the conversion of 2-NP, 3-NP and 2,6-DNP. Figure a SI-9a show the UV-vis spectra of 2-NPs before and after the addition of NaBH_4_. The λ_max_ of the initial solutions of 2-NPs are red shifted from 350 nm to 417 nm, which indicate the formation of 2-nitrophenolate ion. The other peak at 275 nm slightly shifted to 282 nm with the addition of NaBH_4_. Firstly, we introduced Cu/CS-TiO_2_ nanocomposite into the cuvette containing 3 mL of 0.1 mM 2-NP and 0.5 mL of 0.1 M NaBH_4_ solution to investigate its impact on the conversion of 2-NP. Figure 11a exhibited the UV-visible spectral data measured which indicated a gradual decrease in the intensity at the λ_max_ = 417 nm with time. Moreover, the peak at 282 nm was slightly shifted to the 291 nm can be observed indicating the successful conversion of 2-NP to 2-AP. The result obtained strongly support that Cu nanoparticles are responsible for the superior and excellent catalytic activity. In addition to 4-NP and 2-NP, we also investigated the transformation of 3-NP to 3-AP. Figure SI-9b show the UV-vis spectra of 3-NP before and after the addition of NaBH_4_. The λ_max_ of the initial solutions of 3-NPs are red shifted from 330 nm to 390 nm. The conversion of 3-NP was investigated in the presence Cu/CS-TiO_2_ nanocomposite fibers having the same conditions as explained in above text. The results obtained are manifested in Fig. 11b. The UV-vis spectra exhibited that the absorbance at λ_max_ = 390 nm as function of time gradually decreases which suggest the reduction of 3-NP. Similarly, the UV-vis spectra of 2,6-DNP before and after the addition of NaBH_4_ is manifested in Fig. SI-9c. Here also two main peaks were observed for pure solution 350 nm and 430 nm, which indicated the presence of 2,6-DNP. The peak at 350 nm was almost vanished with the addition of NaBH_4_, however, the peak at λ_max_ = 430 nm enhance to high intensity. Figure 11c shows the UV-vis spectra of catalytic reduction of 2,6-DNP as a function of time. The percent reduction calculated by Equation, % Red = (A_0_-A_t_)/A_0_*100 was demonstrated in Fig. 11d. We can observed from the figure comparing percent reduction of nitrophenols in presence of Cu-NPs templated on CS-TiO_2_, that 4-NP was converted faster to 4-AP as compare to other nitrophenols.

#### Mixed dyes and nitrophenols

**Figure 12:**
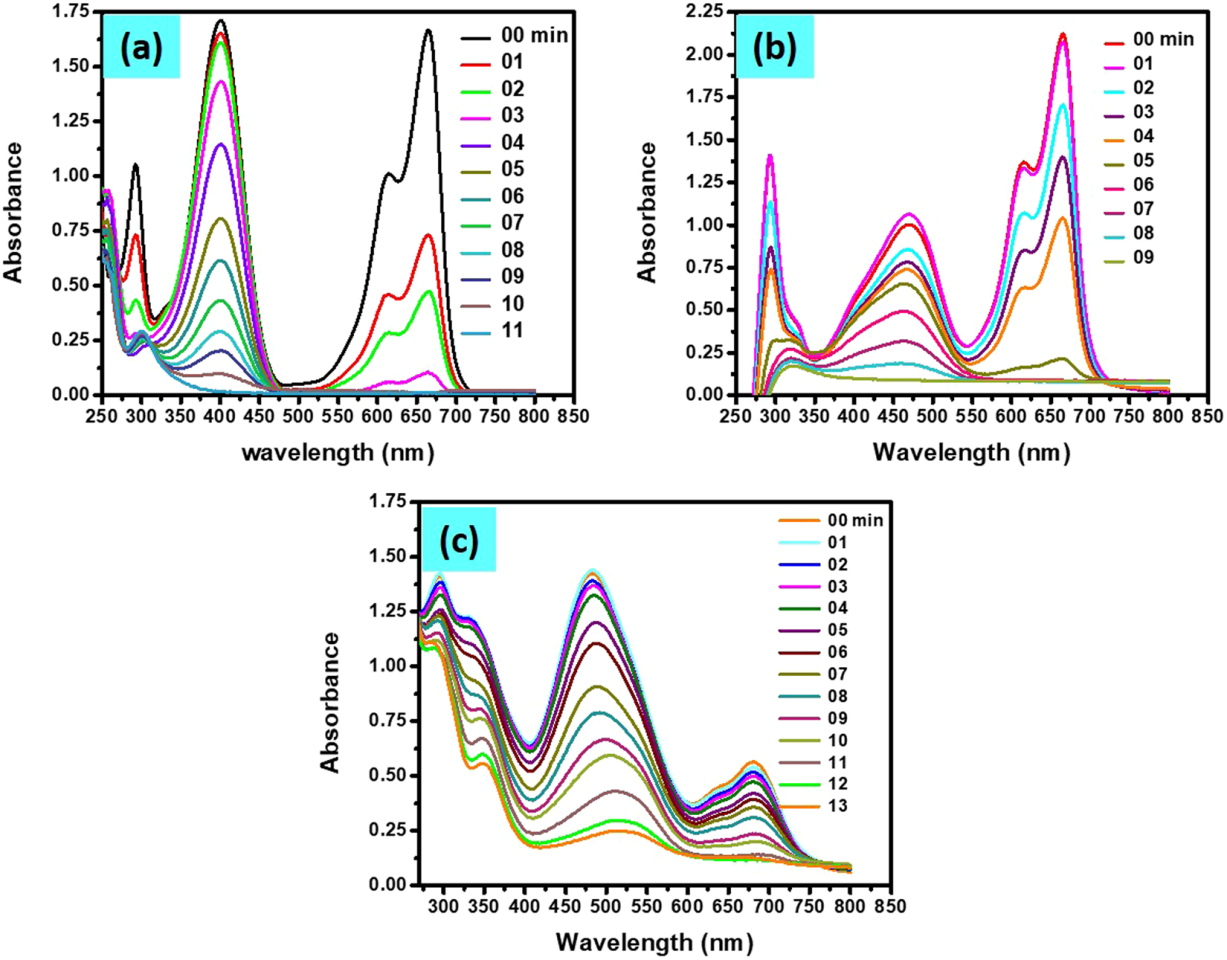
UV-vis spectra of catalytic reduction of mixed solution of 0.1 mM concentration of each 4-NP with MB (**a**), MO with MB (**b**) and CR with MB (**c**) as a function of time by NaBH_4_ in the presence of Cu/CS-TiO_2_ nanocatalyst.

The fast reduction of different dyes and nitrophenols was possible in the presence of Cu/CS-TiO_2_ nanocatalyst. Therefore, to investigate the impact of this catalyst in rear water samples which contain different types of dyes and nitrophenols, we mix dyes and nitrophenols. The UV-vis spectra of mixed solution in the presence of Cu/CS-TiO_2_ nanocatalyst was monitored at constant interval of time. Figure 12a shows the UV-vis spectra of mixed 4-NP and MB solution as a function of time in which two absorbance peaks are distinct, one at 400 nm related to 4-NP and the other 664 nm manifesting the presence of MB in the solution mixture. The regular decrease in both peaks were observed with reaction time. We can see that the peak correspond to MB at 664 nm finish earlier as compared to the other peak which suggest that this material is more active towards MB dye as compared to 4-NP. Similarly, we mixed MB with MO and CR and aspect its catalytic reduction by NaBH_4_ in the presence of Cu/CS-TiO_2_. Figure 12b,c represents the UV-vis spectra of these mixed solutions, respectively. We can observed two peaks from the spectra of MB mixed with MO (Fig. 12b), corresponding at 464 nm and 664 nm confirming the presence of both dyes. Moreover, the peak observed for the solution of MB and CR were at 495 nm and 664 nm, which confirming the presence of both these dyes. The reduction of MB was completed a little bit faster as compared to MO and CR dye. Also the reduction is a little bit slower in case of CR as compared to MO, which might be due to the presence of two -N = N- group in CR as explain earlier.

### Recyclability of the catalyst

It was explicit from the results obtained from the experiments on different dyes and nitrophenols that zero-valent copper (Cu^0^) nanoparticles were mainly responsible for their catalytic reduction. In fact, the catalytic properties of Cu^0^-NPs are widely recognized but the recycling ability are frequently performed by templating them onto substrate. Sometimes even templating onto substrate requires centrifugation^99^. In present study, we prepare the nanocomposite fibers for the uptake of MNPs, where recyclability process was very easy and involved just towing the catalytic fibers from the reaction matrix. Figures SI-10, 11 respectively exhibited the UV-vis spectra recorded during monitoring the catalytic reduction of MO and MB in the presence of Cu/CS-TiO_2_ (re-used) and their percent reduction upto 4-cycles.

**Figure 13:**
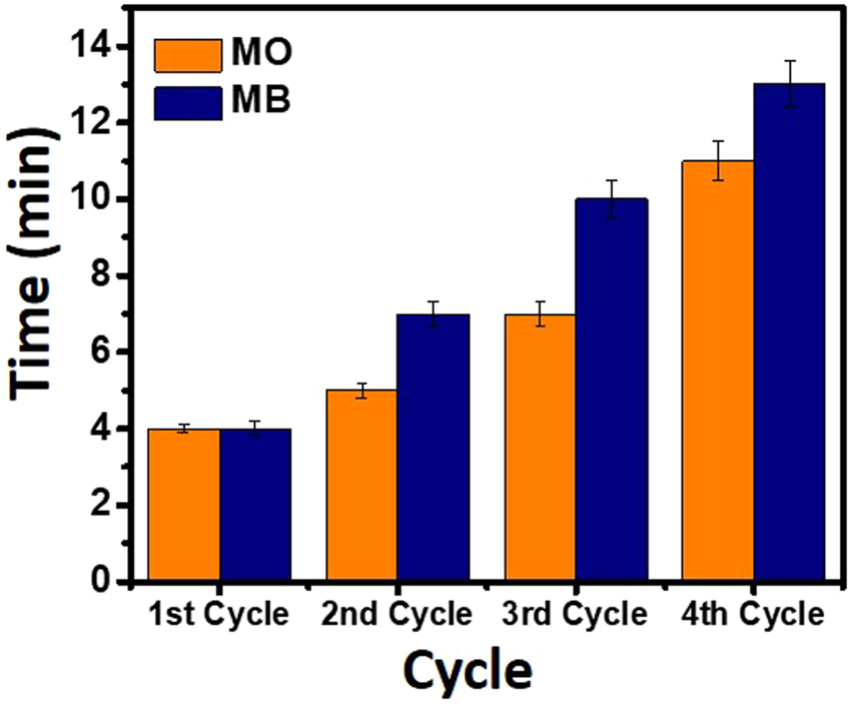
Recyclability of MO and MB showing their complete reduction versus time in the presence of 25 mg of Cu/CS-TiO_2_ (re-used).

The reduction time for the complete reduction of MO during cycles is manifested in Fig. 13. We have observed the increase in the reduction time when the same Cu/CS-TiO_2_ nanocomposite fiber was used, which indicated the decrease in catalytic performance. Such a decrease in the catalytic performance might be due to the oxidation of Cu-NPs during handling and their slight release to the solution matrix.

## Conclusion

We report a simplistic approach to synthesis zero-valent MNPs on a supporting nanocomposite material of chitosan (easily available and biodegradable) and TiO_2_ (less toxic, abundant availability and cheap). The successful preparation of MNPs on the surface of the nanocomposite was confirmed by FE-SEM and EDX analysis. The properties of the chitosan and TiO_2_ did not change much during the process of synthesis of MNPs as revealed by XRD and FTIR analyses. We exhibited that the catalytic properties of Cu/CS-TiO_2_ among all MNPs loaded on various composition of nanocomposite was efficient for the reduction reactions of different organic dyes (MO, CR, MB and AO) and nitrophenols (4-NP, 2-NP, 3-NP and 2,6-DNP). The reduction reactions of MO, CR and MB were completed within a short time of 4 min, moreover AO and nitrophenols (4-NP, 2-NP, 3-NP and 2,6-DNP) were also reduced in less than 15 min, showing its good catalytic ability for all types of organic pollutants. The re-used of Cu/CS-TiO_2_ was investigated for four cycles during the reduction of MO and MB dyes where more than 97% of the reduction was achieved in less than 14 min. The separation of the catalytic fiber was easily performed by simply pulling the fiber from the reaction matrix. The developed approach of preparing MNPs on the surface of CS-TiO_2_ nanocomposite as a substrate has great potential for use in catalytic reaction where high catalytic performance as well as easy separation are required.

## Electronic supplementary material

Supplementary Information
