# Supplementary material for: Chitosan-titanium oxide fibers supported zero-valent nanoparticles: Highly efficient and easily retrievable catalyst for the removal of organic pollutants
Source: Sci Rep. 2018 Apr 19;8:6260. doi: 10.1038/s41598-018-24311-4 (PMC5908960; doi:10.1038/s41598-018-24311-4)
Supplement: Supplementary file 1 — Supplementary Information [file 41598_2018_24311_MOESM1_ESM.pdf]

Supporting informations

Chitosan-titanium oxide fibers supported zero-valent nanoparticles: Highly efficient and easily retrievable catalyst for the removal of organic pollutants

Fayaz Ali[a,b], Sher Bahadar Khan[a,b,*], Tahseen Kamal[a,b,] , Khalid A. Alamry[a], Abdullah M. Asiri[a,b]

[a]*Center of Excellence for Advanced Materials Research (CEAMR), King Abdulaziz University, P.O. Box 80203, Jeddah, Saudi Arabia 21589*
[b]*Department of Chemistry, King Abdulaziz University, P.O. Box 80203, Jeddah, Saudi Arabia 21589*

* To whom correspondence should be addressed. E-mail: sbkhan@kau.edu.sa

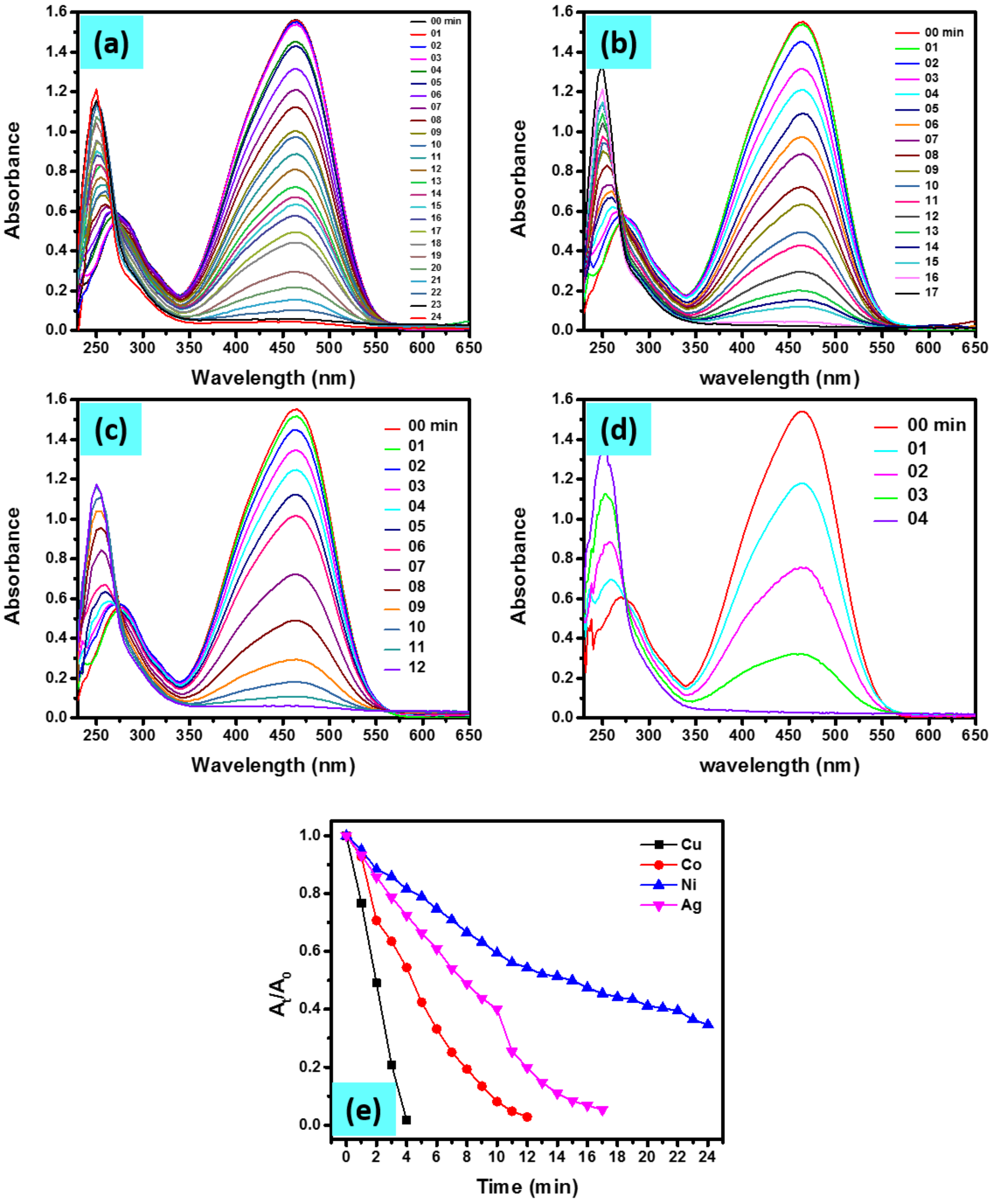

Figure SI- 1 MO dye reduction as function of time by $NaBH_4$ in the presence of CS-TiO_2-15 nanocomposite loaded with Ni (a), Ag (b), Co (c) and Cu (d) nanoparticles, where (e) represents A_t/A_0 versus time of these spectra's.

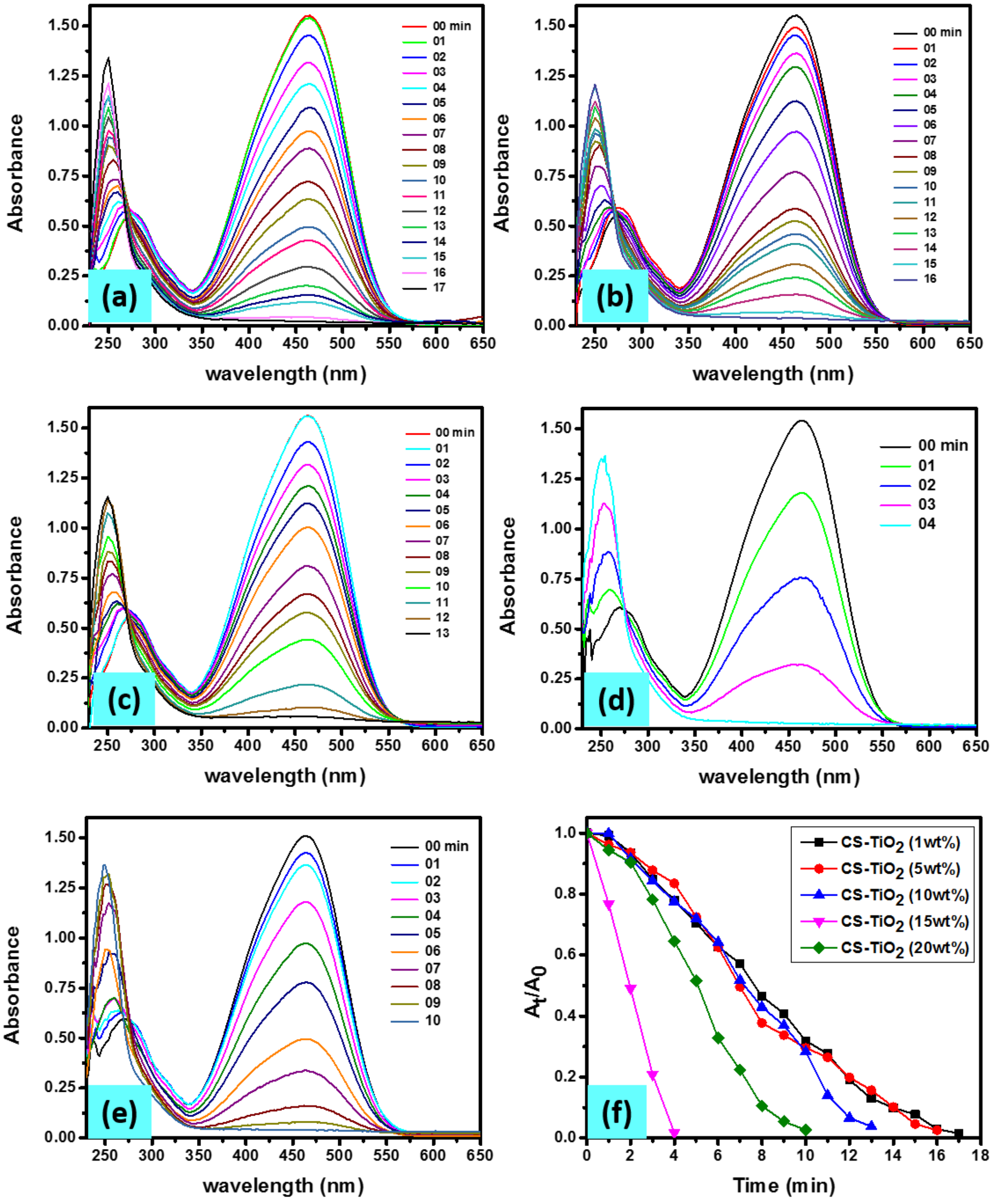

Figure SI- 2. MO dye reduction as function of time by $NaBH_4$ in the presence of Cu nanoparticles templated on CS-TiO_2-1 (a), CS-TiO_2-5(b), CS-TiO_2-10 (c) CS-TiO_2-15 (d) and CS-TiO_2-20 (e), where (f) represents A_t/A_0 versus time of these spectra's.

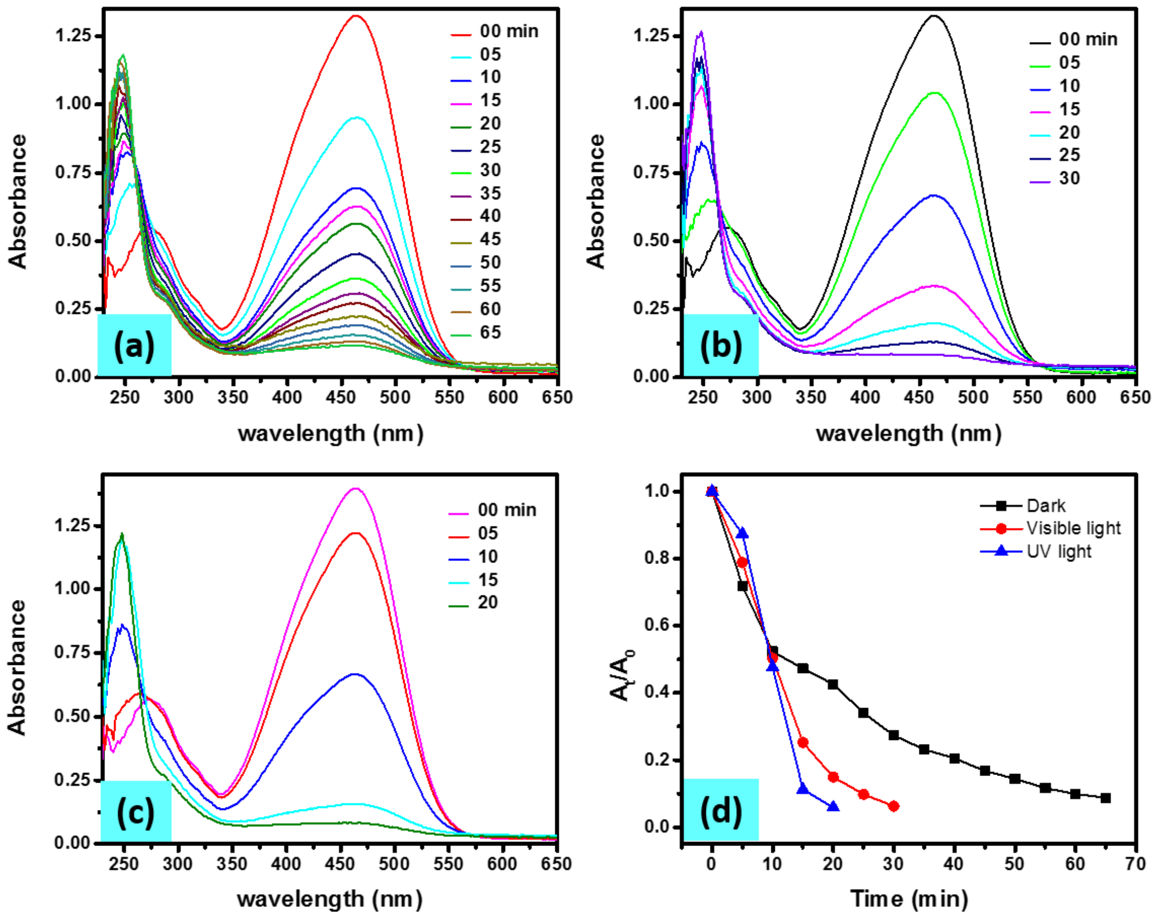

Figure SI- 3 UV-vis spectra of MO dye reduction by $NaBH_4$ as a function of time by using catalyst (Cu/CS-TiO_2) in dark (a) and in the presence of visible light (b), and UV-radiation (c). the ratio of A_t/A_0 verses time of maximum absorbance of these spectra (d).

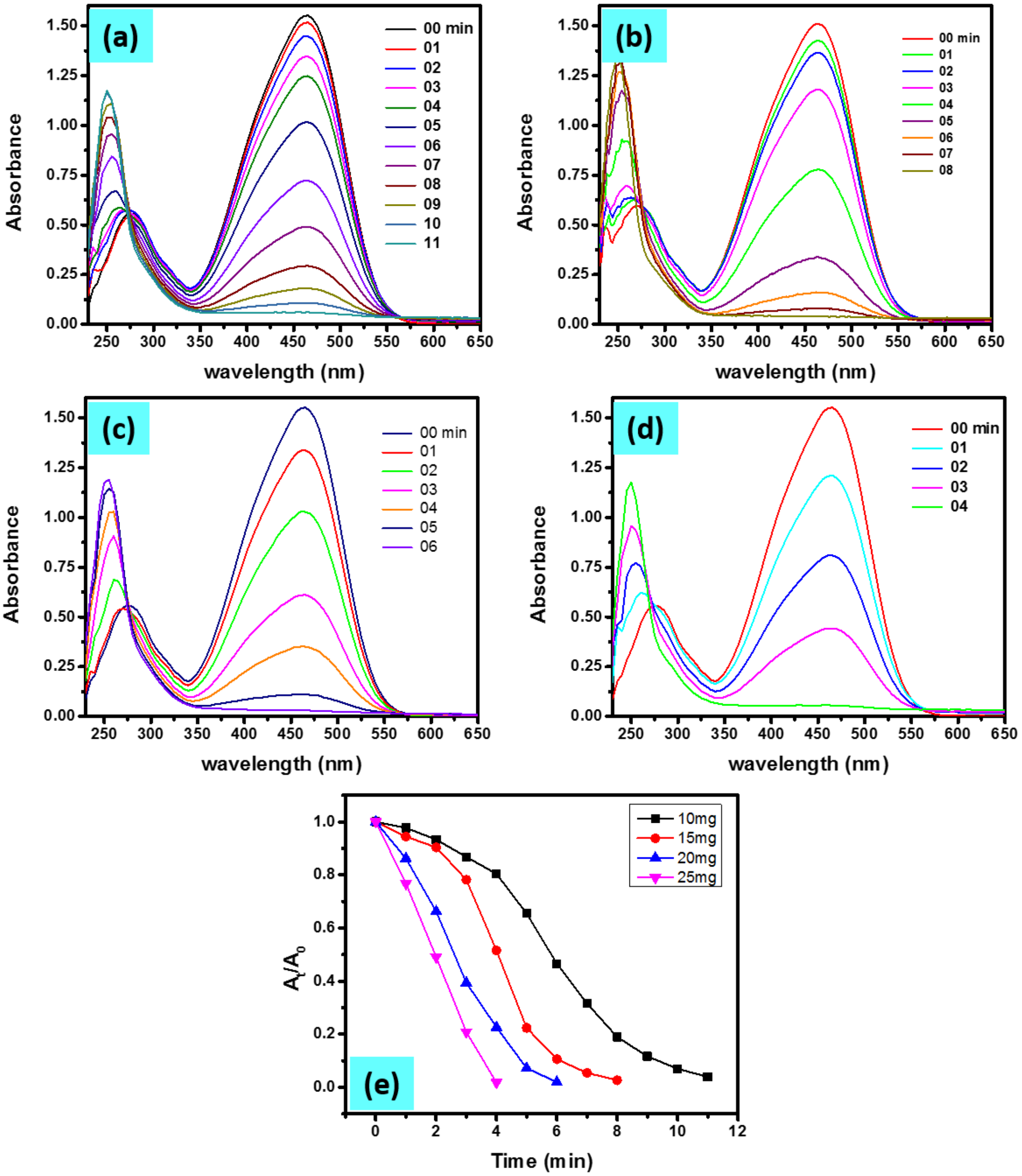

Figure SI- 4 Different amount of catalyst 10 mg (a), 15 mg (b), 20mg (c) and 25 mg (d) Cu/CS-TiO_2-15 used for catalytic reduction of MO by $NaBH_4$.

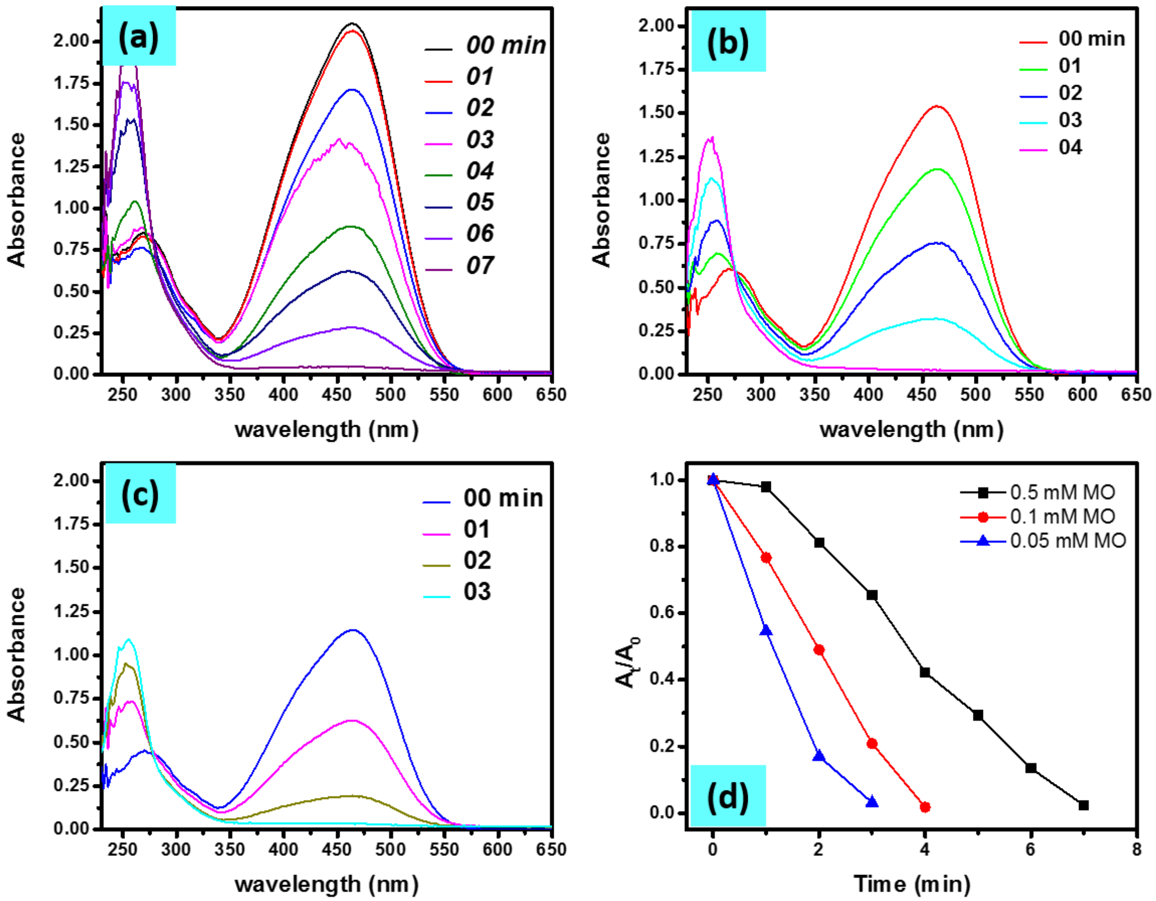

Figure SI- 5 Catalytic reduction of different initial concentration of MO dye by $NaBH_4$ (same amount and concentration) in the presence of $Cu/CS\text{-}TiO_2$ (constant amount)

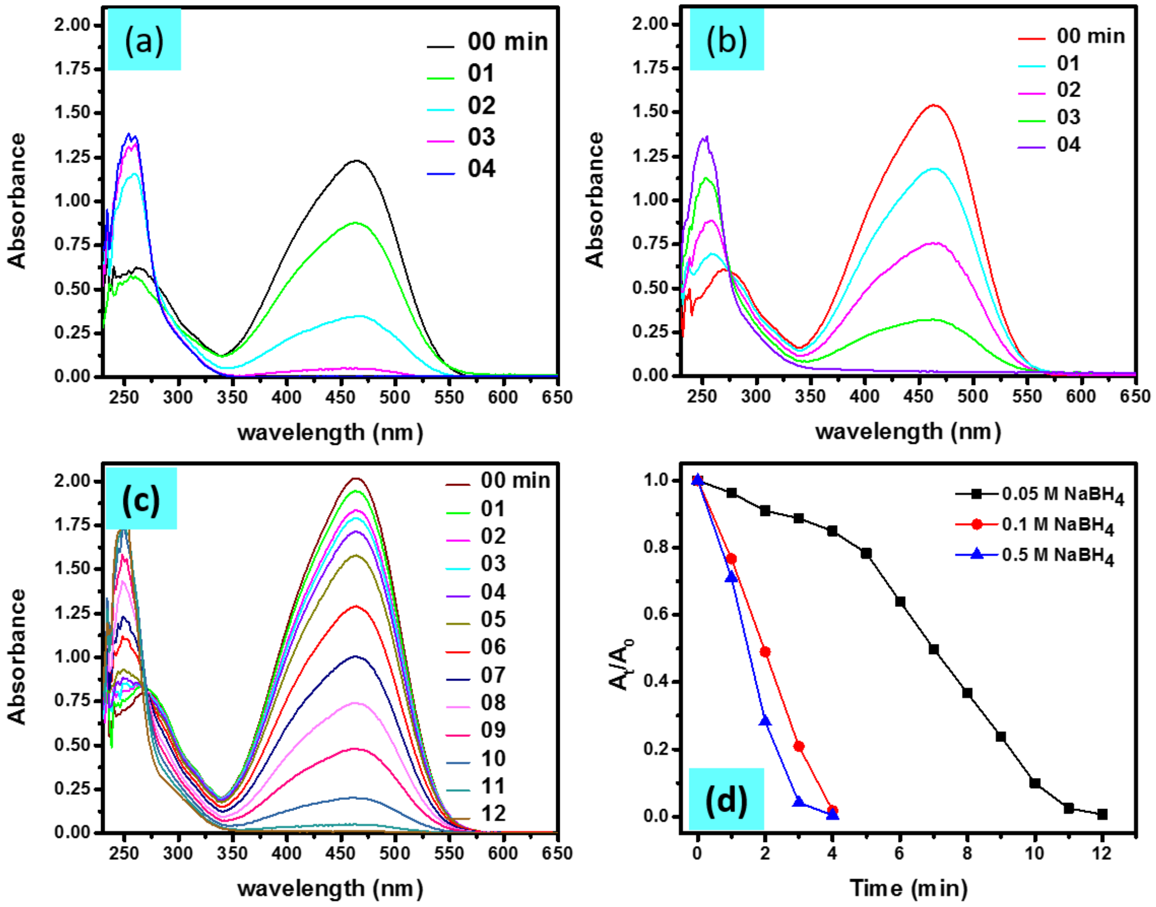

Figure SI- 6 Catalytic reduction of MO by different initial concentration of $NaBH_4$ in the presence of fixed amount of Cu/CS-TiO_2 nanocatalyst.

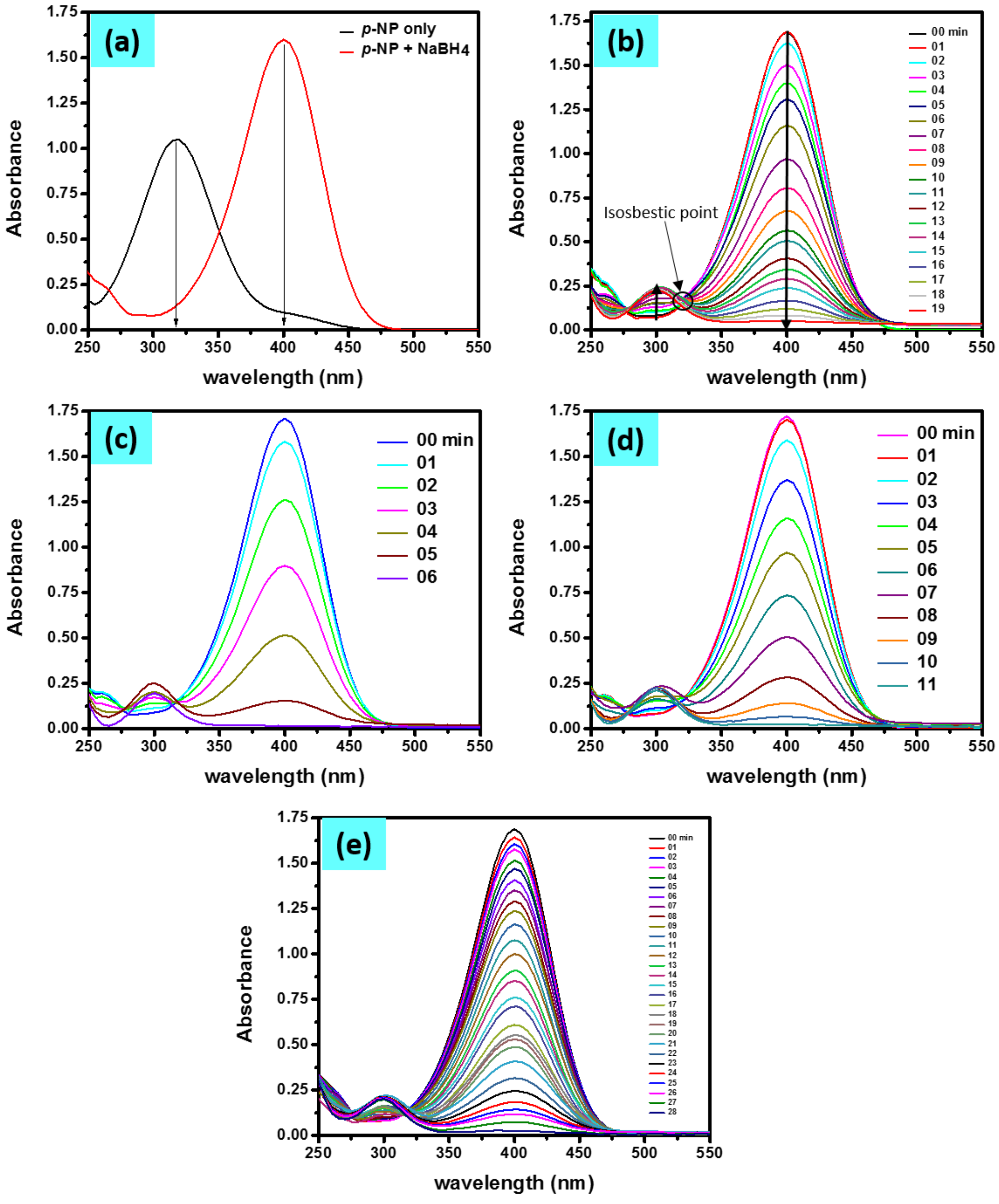

Figure SI- 7 UV-visible spectra of 3mL of 0.1 mM 4-NP before and after the addition of 0.5mL $NaBH_4$ (0.1M) (a), 4-NP conversion to 4-AP in the presence of $NaBH_4$ and CS-15wt%TiO_2 nanocomposite loaded with Ag (b) Cu (c), Co (d) and Ni (e) nanoparticles

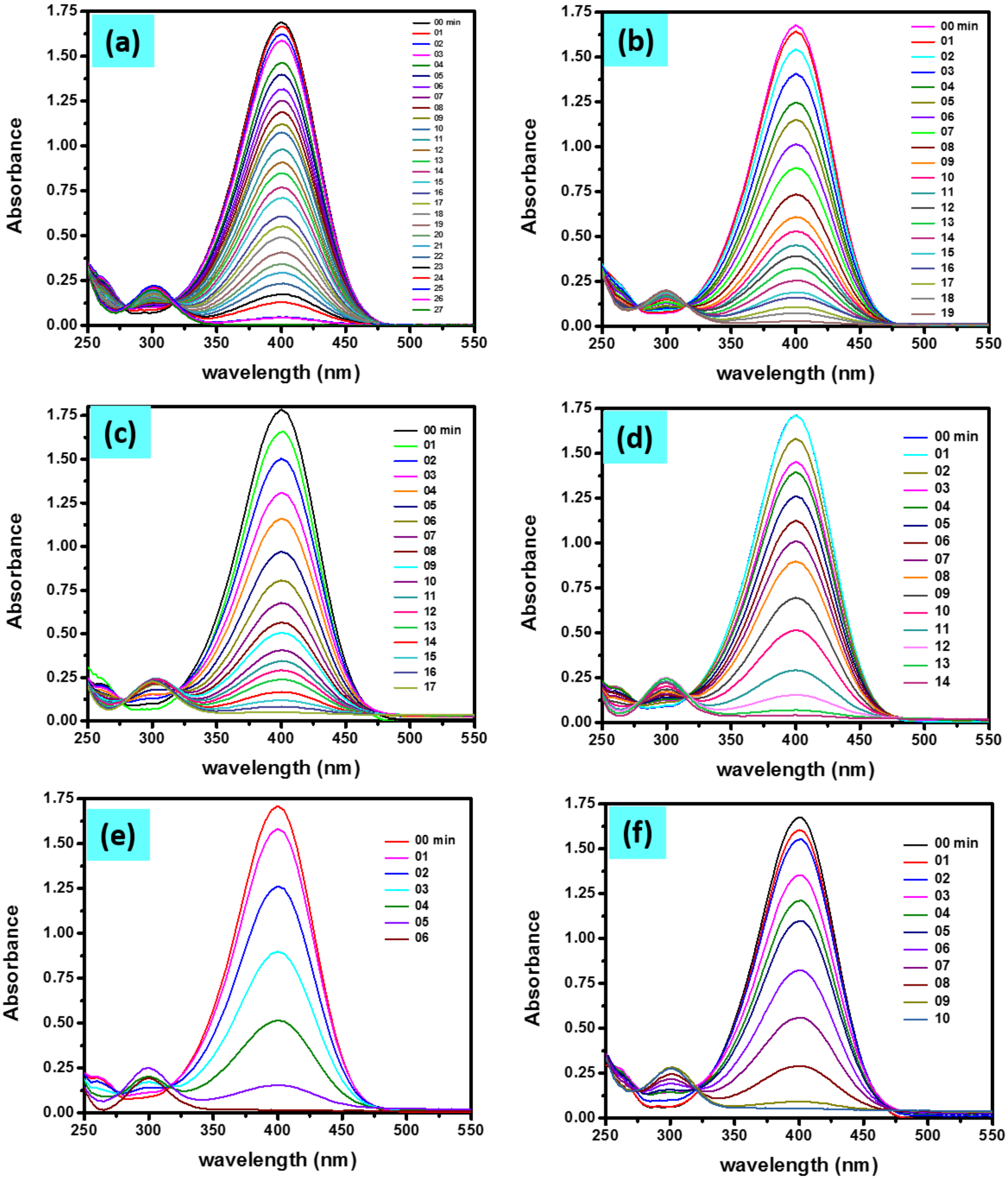

Figure SI- 8 UV-visible spectra of 3mL of 0.1mM 4-NP plus 0.5mL of 0.1M $NaBH_4$ in the presence of catalyst, Cu-NPs templated on pure chitosan fibers (a), and with added TiO_2 of 1wt% (b), 5wt% (c), 10wt% (d), 15wt% (e) and 20wt% (f) nanocomposite.

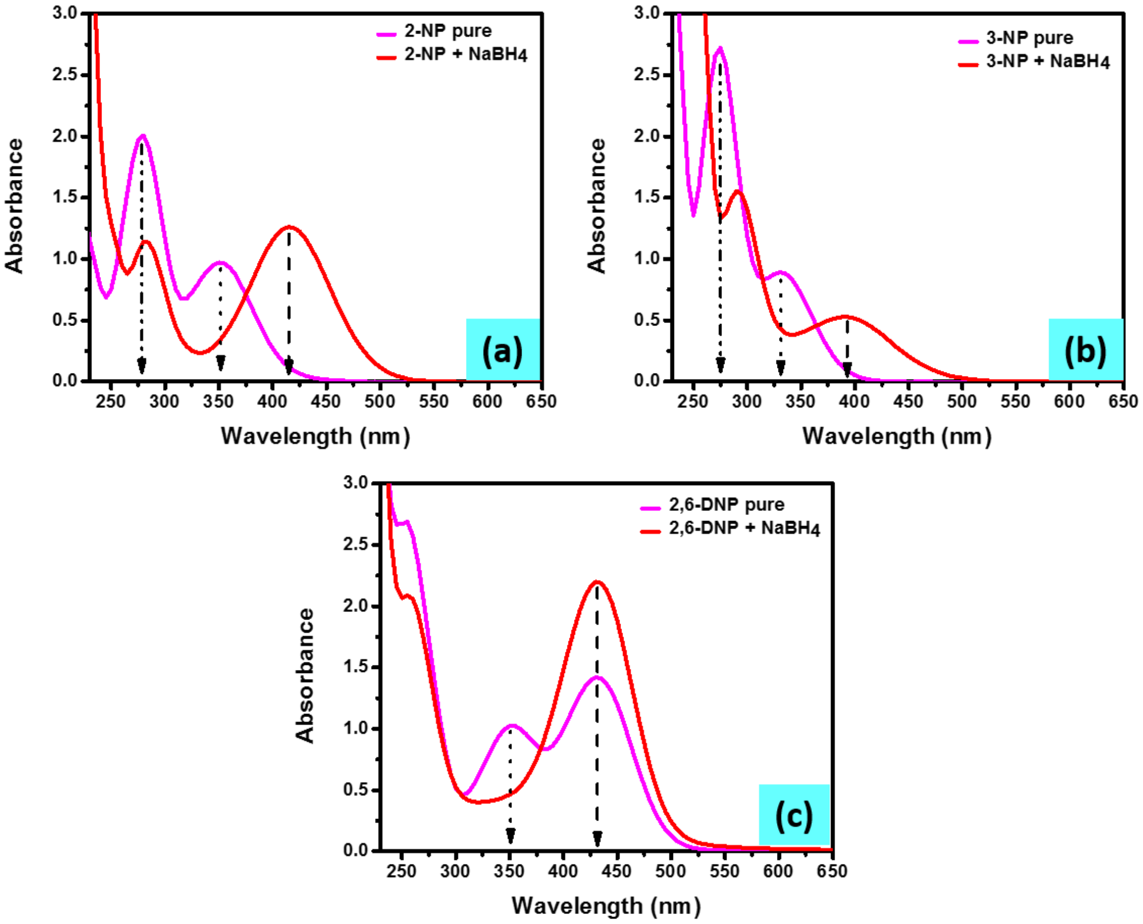

Figure SI- 9 UV-vis spectra of pure and added $NaBH_4$ of 2-NP (a), 3-NP (b) and 2,6-DNP

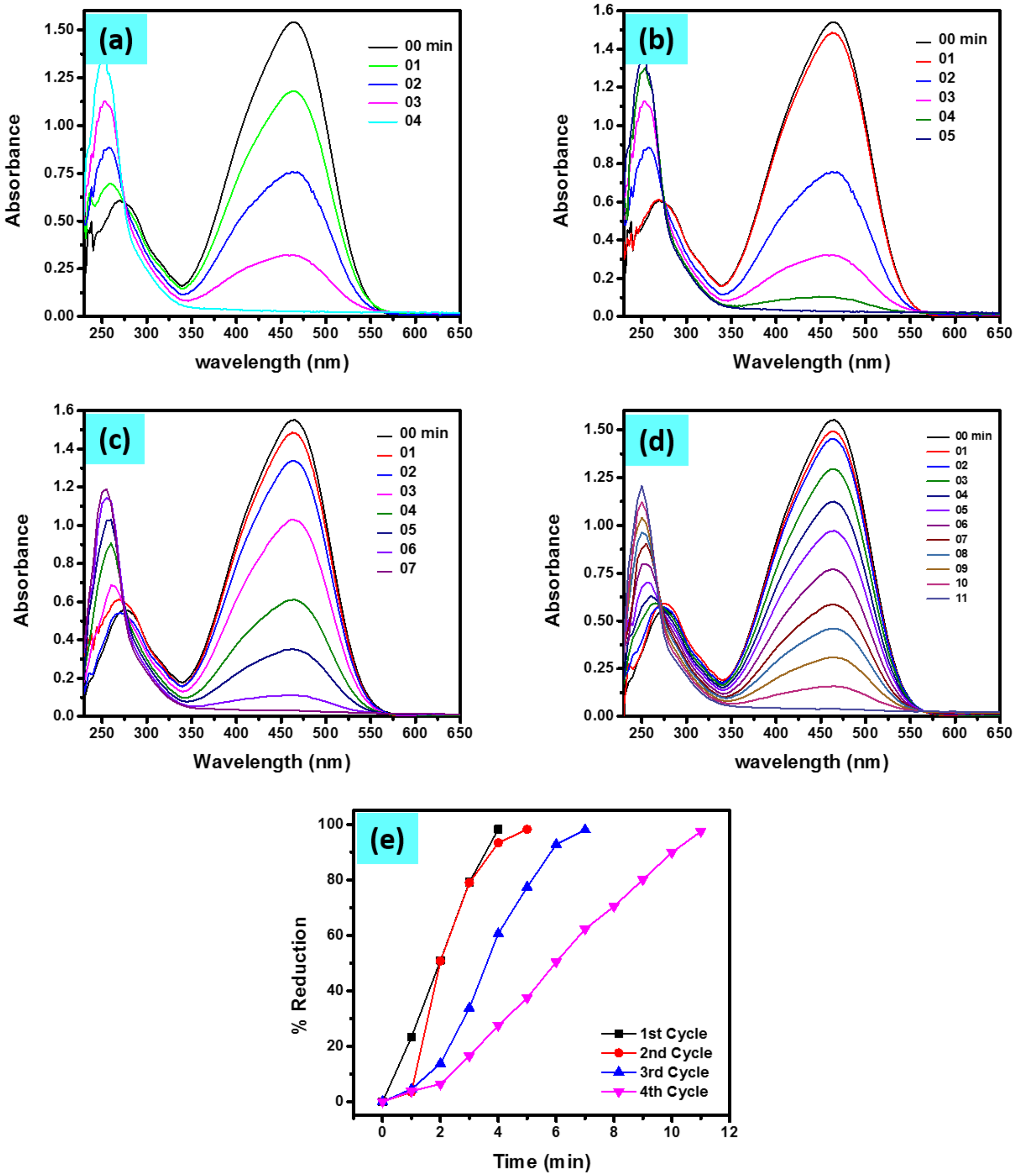

Figure SI- 10 UV-vis spectra of MO reduction by $NaBH_4$ in the presence of same Cu/CS-TiO_2 nanocatalyst used 1st time (a), 2nd time (b), 3rd time (c) and 4th time (d) and comparison of their percent reduction with time (e)

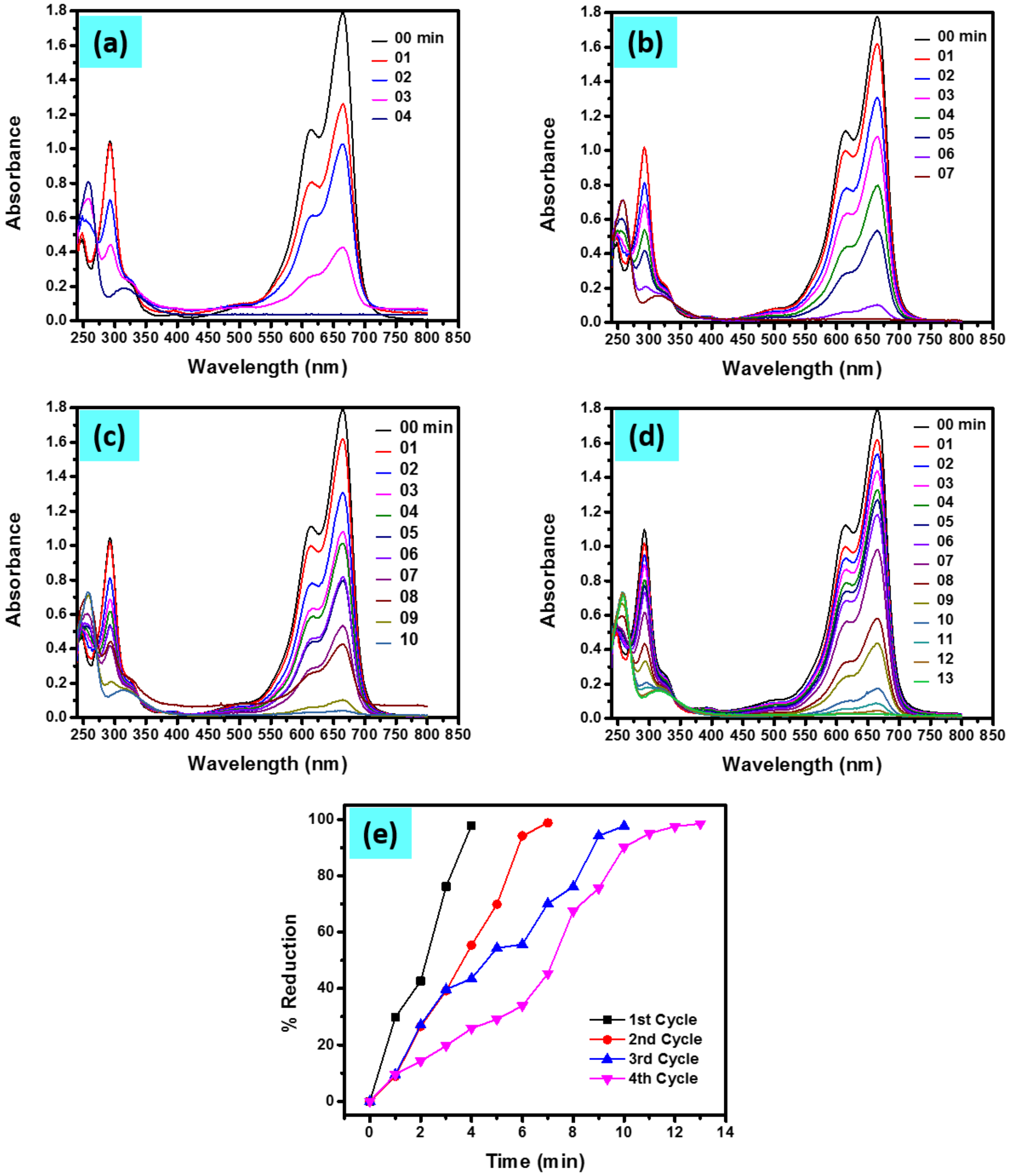

Figure SI- 11 UV-vis spectra of MB reduction by $NaBH_4$ in the presence of same Cu/CS-TiO_2 nanocatalyst used 1st time (a), 2nd time (b), 3rd time (c) and 4th time (d) and comparison of their percent reduction with time (e)
